# Programming structural and magnetic anisotropy for tailored interaction and control of soft microrobots

**DOI:** 10.1038/s44172-023-00145-5

**Published:** 2024-01-05

**Authors:** Yimo Yan, Chao Song, Zaiyi Shen, Yuechen Zhu, Xingyu Ni, Bin Wang, Michael G. Christiansen, Stavros Stavrakis, Juho S. Lintuvuori, Baoquan Chen, Andrew deMello, Simone Schuerle

**Affiliations:** 1 Department of Health Science and Technology, Institute for Translational Medicine, ETH Zürich, Zürich, Switzerland; 2 Department of Chemistry and Applied Biosciences, Institute for Chemical and Bioengineering, ETH Zürich, Zürich, Switzerland; 3 Department of Mechanics and Engineering Science, College of Engineering, Peking University, Beijing, China; 4 National Key Laboratory of General Artificial Intelligence, Peking University, Beijing, China; 5 National Key Laboratory of General Artificial Intelligence, BIGAI, Beijing, China; 6 CNRS, LOMA, University of Bordeaux, Bordeaux, France

**Keywords:** Soft materials, Biomedical engineering

## Abstract

Swarms of soft microrobots controlled by minimally invasive magnetic fields show promise as biomedical agents. The collective behaviour of such swarms, governed by magnetic and hydrodynamic interactions, emerges from the properties of their individual constituents. The introduction of both magnetic and structural anisotropy into microrobots expands the possibilities for tailoring and predetermining interactions and collective behaviours that result. Unfortunately, current methods for large-scale production of soft microrobots, typically result in isotropic properties. Herein, by combining simulation-guided design and droplet-based microfluidics, we present a versatile, high-throughput technique for fabricating soft microrobots with programmable structural and magnetic anisotropy. Such microrobots consist of iron oxide nanoparticles organized into supra-domain structures and entrapped in a hydrogel matrix that can be elongated independently of its magnetic properties. By applying rotating magnetic fields to resulting swarms, distinct collective behaviours are produced, including gas-like formations, variable crystals, and heterogeneous motions.

## Abstract (web summary)

Yimo Yan and colleagues combined droplet-based microfluidics and controlled photopolymerization in an approach to mass produce soft microrobots with programmable structural and magnetic anisotropy. This large-scale production enabled the formation of microrobot swarms and observations of their emergent structures and behaviours.

## Introduction

Microrobots are envisioned to perform biomedical functions, including biosensing and drug delivery. Soft microrobots have garnered particular interest due to their deformability and ability to mimic human blood cells when navigating the cardiovascular system^1,2^. Wireless-transmittable stimuli, such as magnetic fields^3–6^, light^7,8^, ultrasound^9–11^, chemical reactions^12^, or temperature^13^ can be used to control their state and function, such as payload release or propulsion scheme^14^. Magnetic fields are often preferred due to their biocompatibility and excellent tissue penetration. Rotating magnetic fields (RMFs) are particularly advantageous since they can create powerful torques, enabling motion over significant distances within the body^15^. Whilst a single magnetic microrobot cannot normally complete a biomedical task such as drug delivery (More detailed comparisons between microrobot with existing drug delivery systems are shown in Table. S1), swarms of microrobots can be employed^16,17^. Just as the collective motion of animal swarms including flocks of birds emerges from comparatively simple behavioural rules, the response of microrobotic swarms is determined by the properties of individual microrobots^18^. Controlling collective behaviours therefore requires strategies for pre-programming individual microrobots to regulate interactions with others in response to external stimuli. For magnetically responsive soft microrobots in fluids, this principally entails manipulating magnetic dipole interactions and hydrodynamic couplings that arise between neighbours exposed to magnetic fields. By applying dynamic magnetic fields, reconfigurable swarm patterns can be created and controlled^19^. While most swarms consist of microrobots that are identical and symmetrical^20,21^, anisotropic microrobots as well as heterogeneous sub-populations can yield distinct properties and expand function space^22,23^.

Available methods for large-scale production of magnetic microrobots, such as emulsion-based synthesis, typically produce isotropic microrobots, limiting the scope of preprogramed interactions and swarm behaviour. More complex methods, including 3D/4D photolithography^24^ and additive manufacturing^25,26^ yield intricate anisotropic structures for complex control, but suffer from low production rates, high costs, and reduced biocompatibility. (More detailed comparisons among different fabrication techniques are shown in Table S2) Accordingly, methods suitable for large-scale production that break the isotropic symmetry of soft microrobots, either through internal ordering or the introduction of morphological anisotropy^27^, are required. However, effectively combining high-throughput fabrication with the incorporation of distinct interaction behaviours through symmetry breaking continues to pose substantial engineering challenges.

Herein, we present a versatile strategy for designing and mass-producing soft microrobots with programmable structural and magnetic anisotropy using droplet-based microfluidics and photopolymerization guided by external magnetic fields. We integrate computational modelling and simulation-guided design to tailor microrobot anisotropy, and control their response to external magnetic torques. By tuning hydrodynamic and magnetic interactions, we generate multiple distinct collective behaviours, including gas-like, variable crystal, and heterogeneous motions. We complement experimental data with numerical simulations to determine key design principles that drive the physical mechanisms underlying swarm behaviour. We offer a design/fabrication pipeline for soft microrobots with distinct structural and magnetic anisotropy that improves our understanding of the basic principles of swarm behaviour.

## Results

### Design and manufacture of microrobots with distinct anisotropy

**Fig. 1:**
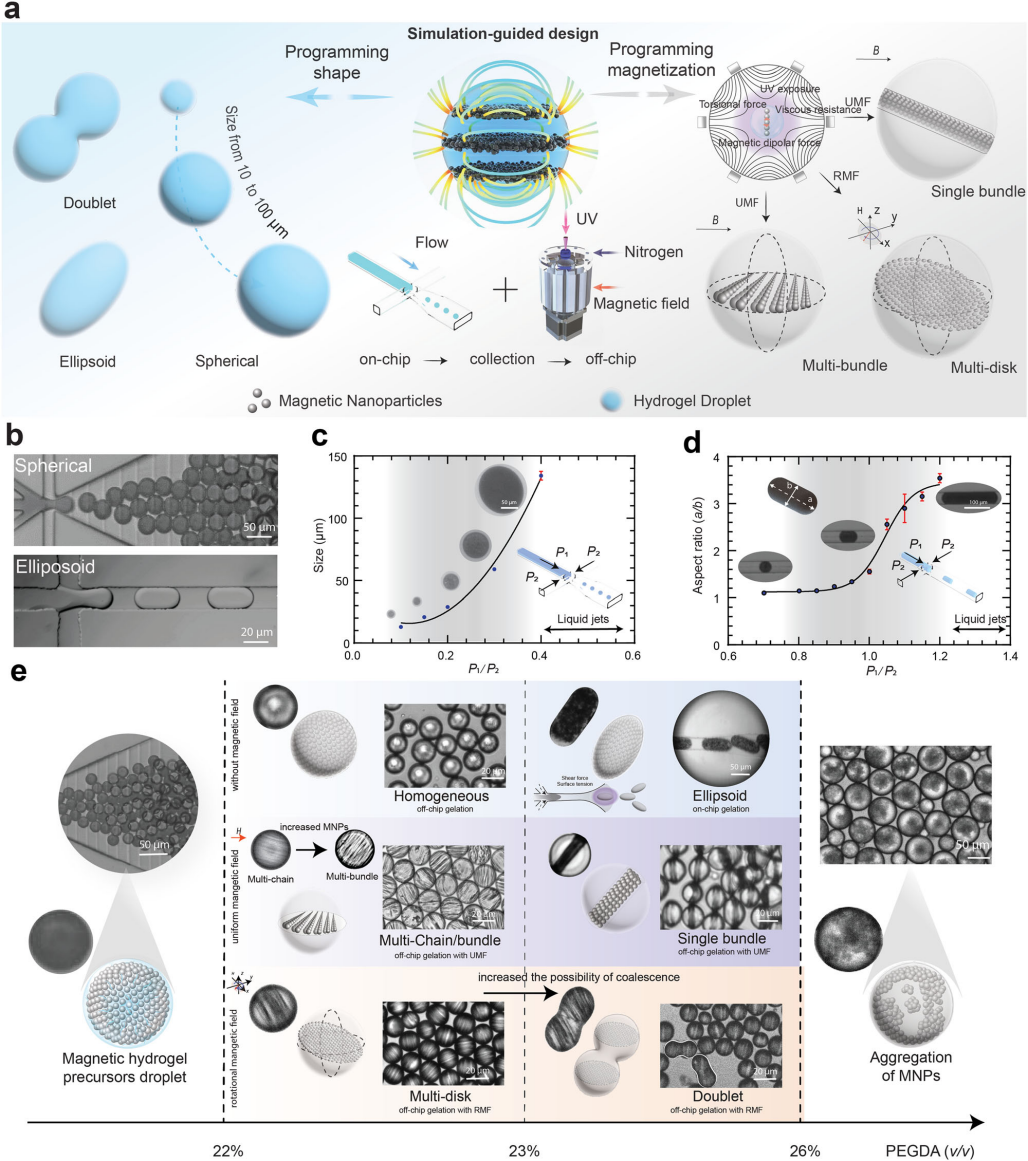
Programming and simulation-guided fabrication of soft microrobots with structural and magnetic anisotropy. **a** Schematic of the integrated approach to program structural and magnetic anisotropy in soft microrobots. **b** Controllable fabrication of precursor droplets with variable structural anisotropy (e.g., sphere, ellipsoid) can be achieved using high throughput droplet-based microfluidics. **c** The size of spherical microrobots can be controlled by varying applied pressure ratios between the discrete and continuous phase. As ${P}_{1}/{P}_{2}$ increases, the size of droplet also increases. Error bars represent the SD of the mean. **d** Aspect ratio of ellipsoidal microrobots and thus, their structural anisotropy, can be varied by controlling applied pressure ratios. As ${P}_{1}/{P}_{2}$ increases, the aspect ratio ($a/b$) of droplet also increases. Error bars represent the SD of the mean. **e** By adjusting volume fraction of Polyethylene glycol diacrylate (PEGDA), implementing different gelation methods (on- vs. off-chip) and varying the magnetic field stimuli, multiple microrobot shapes and distinct magnetic supra-domain structures can be realised, as depicted in the fabrication phase diagram of anisotropic soft microrobots. The confocal microscope image displays the microrobot products, with their distinct magnetic supradomain schematically depicted alongside. (More details of fabrication are shown in Fig. S9).

To generate programmable and biocompatible magnetic microrobots that imbue structural and magnetic anisotropy, hydrogel and magnetic nanoparticles (MNPs) were chosen as core constituents. We aimed to fabricate doublets, ellipsoids, and spheres with internal magnetic supra-domains composed of chains, disks, bundles, and homogenously distributed MNPs (Fig. 1a).

To guide fabrication, we developed an a priori particle-based simulation model to analyse and predict magnetization and magnetic interactions of soft microrobots with different structural and magnetic anisotropies. Followingly, we used droplet-based microfluidics integrating controlled photopolymerization (Fig. S1a) under static, rotating, or no magnetic fields. First, magnetic hydrogel precursor droplets are generated at a flow-focusing geometry (Fig. S1b, Fig. S1b and S1d). The volumetric flow rate controls both the size and stability of the precursor droplet, with the channel design and dimensions determining the shear forces that control droplet shape (Fig. 1c). Free-radical polymerization crosslinking is triggered by UV irradiation and fixes microrobot structure. For on-chip gelation^28,29^, precursor droplets are confined in a narrow microchannel and retain their aspect ratio after photopolymerization, providing structural and magnetic shape anisotropy (Fig. 1d, Supplementary Video 1 and Fig. S1c and S1d). For off-chip gelation, photopolymerization is performed inside a Halbach cylinder providing a static uniform magnetic field (UMF) or RMF (Fig. S2). When using a static UMF, MNPs will arrange into a multi-chain configuration within the droplet due to dipolar interactions. (Fig. S2a) As MNP concentration increases, thicker bundled structures form. Under 5 Hz RMFs, chains of spinning MNPs generate localized fluid vortexes, which lead to the formation of a multi-disk pattern. (Fig. S2b) Upon assembly, structures are stabilized through photopolymerization. Depending on the ratio of MNPs and Polyethylene glycol diacrylate (PEGDA) and the type of magnetic field, different microrobot structures are generated, incorporating the aforementioned magnetic supra-domains (Fig. 1e).

### Encoded magnetization controls the applicable torque and interactions between entities in assemblies

**Fig. 2:**
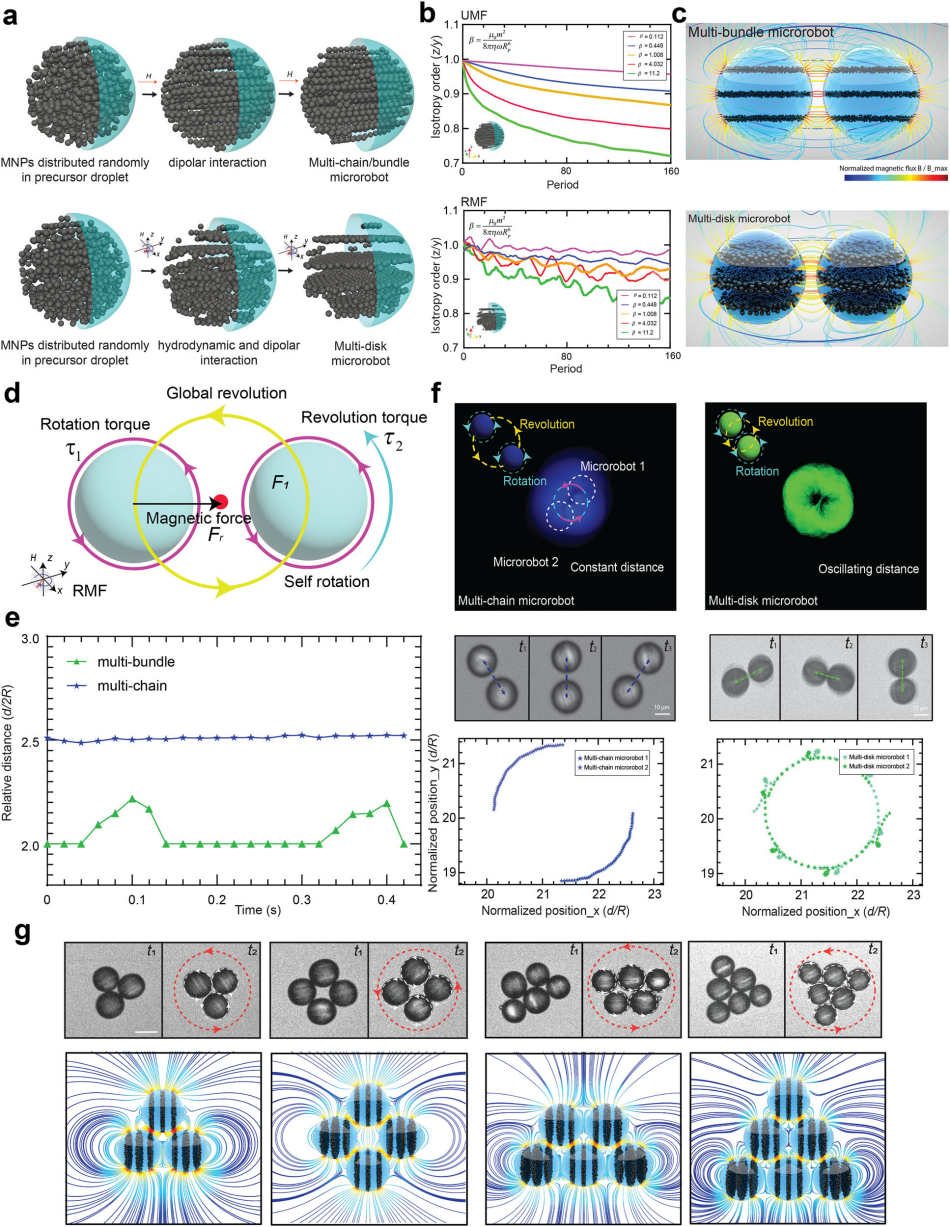
The role of magnetic anisotropy in individual, paired, and multiple microrobot systems. **a** Simulation of magnetic nanoparticles (MNPs) assembly within a precursor droplet under a uniform magnetic field (UMF) and a rotating magnetic field (RMF). **b** Isotropy order of magnetic domains under a UMF and RMF using various magnetic field strengths. **c** Magnetic flux lines demonstrate how engineering the magnetic anisotropy of individual microrobots (Multi-bundle and Multi-disk) can enable tailoring of their magnetic interactions within paired systems that serve as the basic unit of a swarm. **d** Schematic of the interactions associated with a microrobot pair. **e** The timewise evolution of the distance between pairs of microrobots of different magnetic anisotropy (multi-chain, multi-bundle) under an external RMF of 20 mT and 20 Hz. **f** Assembly and interaction processes of microrobot pairs with different magnetic anisotropies shown through experimentation. By applying RMFs to the indicated microrobot pairs, distinct trajectory behaviours are produced, including revolution with constant relative distance (multi-chain microrobots revolve around a central point with a constant distance), revolution with oscillating relative distance (back-and-forth relative motion of multi-disk microrobots). **g** Assembly of multi-disk microrobots of varying number. Stable assemblies of multi-disk microrobots were formed from dimers to hexagonally ordered structures under an RMF (Scale bar: 25 µm). The simulated magnetic flux of the respective clusters is depicted.

Patterned magnetic supra-domains determine a microrobot’s magnetic anisotropy and influence interactions between individual entities within assemblies. Using particle-based simulations, we aimed to capture the magnetization process and predict resulting collective behaviours. To model the assembly process, we confined assembly to a spherical area in which MNPs are suspended in a Newtonian fluid and exposed to magnetic stimuli (Fig. 2a and Supplementary Video 2). When applying a UMF, MNPs preferentially arrange into chain/bundle structures, whilst under an RMF, they display a predisposition for disk structures. As magnetic fields increase, a faster growth of magnetic anisotropy and lower isotropic order are observed (Fig. 2b).

We next analysed the effective torque upon exposure to external fields, testing the influence of magnetic anisotropy on torque-based locomotion of individual microrobots by comparing the torque generated at varying aspect ratios of the magnetic supra-domain under an RMF (Fig. S3a). As expected, for fixed MNP concentrations, effective torque increased with aspect ratio, as it results in a reduction in demagnetization factor, yielding a stronger magnetic dipole interaction between individual MNPs. Analogously, we utilized the same amount of MNPs in both multi-disk and homogeneous microrobots, which led to a higher step-out frequency for multi-disk patterned microrobots due to strong magnetic anisotropy, as demonstrated experimentally and in silico (Fig. S3b).

Next, we studied the interaction between pairs of identical microrobots (Fig. 2c, d) starting with multi-chain microrobots (4% (*v/v*) MNPs). Here, dipole-dipole interactions are weak compared to hydrodynamic interactions ($\beta \approx 0.06$, based on experiments), with the distance between two multi-chain microrobots being constant (Fig. 2e). Multi-chain microrobots are advected by rotational fluid flow created by their partner, leading to rotation around a central hydrodynamic origin (Supplementary Video 3). Indeed, it has been shown via numerical simulations that within a Stokes flow, particle pairs without dipolar interaction will follow a circular path around each other^30^.

Increasing magnetization enhances dipolar interactions between microrobots. Multi-disk microrobots contain a higher concentration of MNPs than microrobots with multi-chains, thus, their dipole-dipole interaction is stronger (10% (*v/v*) MNPs, $\beta \approx 14$). Here a spiral trajectory is observed, a pattern that originates from a forward, stop, and backward motion, as magnetic forces are insufficient to overcome fluidic drag, and thus revolution becomes asynchronous. This microrobot configuration rotates faster than the dimer, which breaks the rigid body configuration. The phase lag between dipole direction and the pair connection changes periodically. Radially, microrobots alternatingly attract and repel each other, leading to an oscillation in the inter-distance (Fig. 2e, f), and transversely, the magnetic force also periodically drives forward and backward revolution. Interestingly, the forward transverse force occurs at a shorter inter-distance, resulting in a stronger forward revolution than backward revolution. Meanwhile, the rotational fluid flow continuously provides a forward revolution. These factors result in spiral forward revolution for moderate magnetic interaction regimes (Supplementary Video 3).

We next sought to investigate the assembly of individual microrobots by introducing more close neighbours. The snapshots in Fig. 2g report the assembly of multi-disk microrobots at low frequency (~5 Hz) from rotating dimers to trimers, quadrilaterals, trapezoids, triangles, and eventually stabilized hexagons (hydrodynamic simulations of clusters are shown in Fig. S4 and Supplementary Video 4). These dynamic steady states result from the combined effect of orientation-dependent magnetic and hydrodynamic interactions. We simulated the magnetic interaction and calculated force and torque within the system to elucidate underlying mechanisms. Specifically, we calculated the rotation and revolution torque of the corner (pink) and center (blue) microrobot inside the hexagon cluster (Fig. S5). To only reflect the response of the external field to microrobot interactions, we selected the characteristic quantity ${m}_{0}{H}_{0}$ to represent the torque; where ${H}_{0}$ is the modulus of the applied external magnetic field, ${m}_{0}=4\pi {\mu }_{0}{a}_{0}^{3}{H}_{0}$ is the modulus of the magnetic dipole moment excited by ${H}_{0}$ of a single microrobot in the limit of infinite relative magnetic permeability, and ${a}_{0}$ is the microrobot radius. Findings demonstrate that multi-disk microrobots at corner positions exhibit heightened proclivity for rotation and revolution. This endows multi-disk microrobots with the ability to induce rotational and translational motion simultaneously within a hexagon cluster. These results indicate that locomotion and magnetic interactions can be regulated by adjusting magnetic anisotropy and magnetization of the entities, leading to control of assembly behaviour.

### Effects of structural and magnetic anisotropy govern separation dynamics in homogenous and mixed swarm systems

**Fig. 3:**
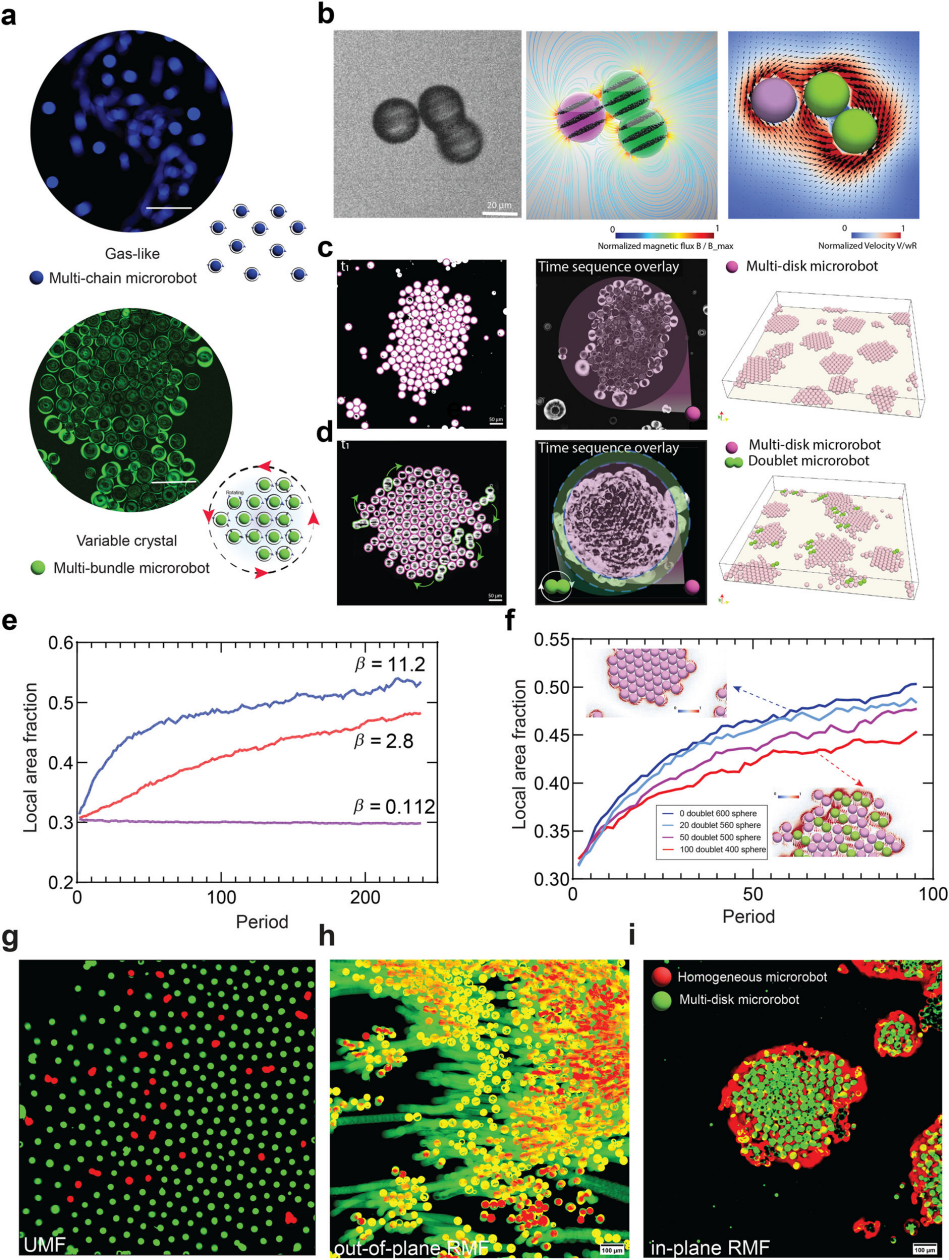
The synergistic impact of structural and magnetic anisotropy on swarm behaviour. **a** Achievable collective behaviours that are tailored by programmable magnetic anisotropy under Rotating magnetic fields (RMFs), including gas-like (multi-chain microrobots), variable-crystal (multi-bundle microrobots) formations. (Scale bar: 100 µm) **b** Experimental images of the assembly and interaction of structurally anisotropic (doublet microrobot) and magnetically anisotropic (multi-disk microrobot) microrobots driven by an RMF at 2 Hz. The simulated flow and magnetic field during the assembly process are shown. **c** Formation of a variable crystal structure composed of spherical (shape isotropic) microrobots under the same RMF input. The time sequence overlay depicts the motion patterns of multi-disk microrobots, effectively representing their movements within a specified time interval. **d** Phase separation process for a mixed swarm system. Over time, the doublet microrobots undergo a progressive segregation from the central region of swarm, leading to their eventual transition into “border walkers” positioned at the periphery of the crystal. **e** The local area fraction versus time for isotropic microrobots under different magnetic field strengths. The rate of aggregation accelerates, while the local area fraction intensifies, as a result of stronger magnetic interactions arising from increased magnetic field strength **f** The local area fraction versus time for increasing concentrations of shape anisotropic microrobots. Elevated concentration ratios of doublet microrobots elevate the effective noise of the system, thereby amplifying disruption to the aggregation process and reducing the local area fraction. **g**, **h** and (**i**) The mixed swarm system consists of homogenous (magnetically isotropic) and multi-disk (magnetically anisotropic) microrobots and is subject to magnetic stimuli, resulting in heterogeneous control under the same magnetic stimuli. By capitalizing on the inherent variability in step-out frequencies among distinct microrobots, it is feasible to independently control specific subpopulations.

Having analysed magnetic interactions for pairs of identical microrobots, we next examined the collective dynamics of microrobot swarms (Fig. 3a). Upon exposure to dynamic magnetic fields, distinct forces and torques induce motion as a result of interactions with neighbouring entities and their surroundings. We distinguish two key behaviours: gas-like and variable crystal based on collective motion patterns exhibited by microrobot swarms, as well as interdependent movement behaviours amongst individuals within the swarm. Hydrodynamic and magnetic interactions are regulated through individual magnetic supra-domains, with a decreasing concentration of encapsulated MNPs weakening magnetic interactions. For multi-chain microrobots, below a threshold MNP concentration, dipole-dipole interactions become negligible and will only have a weak influence on collective behaviour, whilst hydrodynamic interactions will dominate, resulting in a ‘gas-like’ nature under RMF. In contrast to multi-chain microrobots, multi-bundle microrobots have higher concentrations of MNPs and stronger magnetic interactions. The aligned pattern of magnetic supra-domains enables anisotropic magnetic interactions. Combined with moderate magnetic interactions, individual microrobots may exhibit self-rotation around their axis, in addition to global rotation of the whole assembly, i.e., ‘variable crystal’ behaviour. This analysis highlights how microrobot design, guided by magnetic interactions, governs dynamic self-assembly and collective behaviour, yielding multiple collective modes (Supplementary Video 5).

Swarm behaviours driven by RMFs are sensitive to structural anisotropy. Accordingly, we studied doublet and spherical microrobots coupled to an RMF, to evaluate the combined effect of structural and magnetic anisotropy on assembly and locomotion. Given that any pair forms the cornerstone of swarm systems, we initially analysed the behaviour of two neighbouring microrobots of different shape in the presence of an RMF. Microrobots interacted magnetically and hydrodynamically, resulting in the formation of an unsteady spinning pair. For structurally anisotropic, doublet microrobots, short-range repulsion will prevent microrobots from getting too close. We next performed simulations of fluid and magnetization dynamics of this uneven paired system (Fig. 3b, Fig. S6, and Supplementary Video 6). Both show that when spinning microrobots approach each other, they hydrodynamically interact whilst rotating and revolving around each other. However, experimentally, this hydrodynamic interaction is partially overcome by strong dipolar interactions between microrobots. Thus, a spherical microrobot can become temporarily trapped within the hydrodynamic field of a doublet. By controlling magnetization, magnetic interactions can be regulated, ultimately impacting collective behaviour.

We next investigated collective dynamics in a larger, mixed swarm system. Under an RMF, spinning spherical microrobots are subject to magnetic dipolar forces, which cause them to attract and trap adjacent microrobots. Initially randomly distributed microrobots, spontaneously assembled into ordered arrays (close-packed hexagonal structures) forming a rotating variable crystal (Fig. 3c and Fig. S7). The introduction of doublet microrobots into the swarm introduces structural anisotropy, leading to the disruption of crystal-like aggregation associated with structurally isotropic microrobots (Supplementary Video 7). Over time, such doublet microrobots gradually separate from the center and ultimately morph into “border walkers” at the crystal edge (Fig. 3d); a process well captured by simulation (Fig. 3d and Supplementary Video 7). The influence of magnetization on separation dynamics of mixed swarm systems was evaluated by measuring local area fractions as a function of time for various magnetic strengths ($\beta \approx 0.1$, $2.8$ and $11.2$). Aggregation becomes faster, with the local area fraction increasing as the magnetic strength increases due to stronger magnetic interactions (Fig. 3e). However, when the concentration ratio of doublet microrobots increases, effective noise rises, causing disrupted aggregation, and resulting in a smaller local area fraction (Fig. 3f).

Next, we studied the locomotion of heterogeneous swarms comprising spherical microrobots with different magnetic anisotropies but identical structural anisotropy. Figure 3g–i presents experimental results of a heterogeneous swarm containing both multi-disk and homogenous microrobots, illustrating the impact of magnetic anisotropy on separation dynamics. Under a UMF in the *z*-direction, magnetic anisotropy causes microrobots to repel each other, resulting in a uniform distribution (Fig. 3g and Supplementary Video 8). Next, we applied an out-of-plane RMF with a frequency of 20 Hz. This is above the step-out frequency for homogenous microrobots, but below the frequency for multi-disk microrobots. Accordingly, multi-disk microrobots are able to maintain stable rolling, whilst homogeneous microrobots step out and remain in their original position (Fig. 3h and Supplementary Video 8). Under a 20 Hz in-plane RMF, multi-disk microrobots are still able to spin, whereas homogenous microrobots cannot constantly move and undergo asynchronous motion. Interactions between microrobots within a swarm leads to a phase separation phenomenon whereby homogenous individuals are excluded towards the swarm border. Despite being located at the perimeter, these homogenous microrobots continue to maintain magnetic interactions with other entities within the swarm, which results in their unique “border walking” behaviour. Notably, the locomotion ability of border walkers is not solely derived from the torque generated by the external magnetic field but also arises from magnetic and hydrodynamic interactions with adjacent microrobots. (Fig. 3i and Supplementary Video 8). These scenarios confirm the importance of understanding and predicting microrobot behaviour with distinct structural and magnetic anisotropy and its usefulness for the control of microrobotic swarm systems.

## Discussion

Robust fabrication of bespoke soft microrobots is important for advancing our understanding and application of microrobotic technology, with the optimization of both individual and swarm behaviour being critical in engendering biomedical application. This work introduces simulation-guided design of soft microrobotic systems, incorporating droplet-based microfluidics and polymerization under static and dynamic magnetic fields. Such an approach allows the production of monodisperse microrobots with distinct structural and magnetic anisotropy, facilitating improved locomotion and control of swarm dynamics.

The complex and variable physiological environments require microrobots to be customized according to specific needs and desired tasks. Accordingly, the ability to program collective behaviours is of paramount importance. For instance, in drug delivery, microrobot properties will vary depending on the targeted vessels. In larger veins, where flow rates are typically high, it is essential for microrobots to exhibit robust locomotion capabilities. Formation of clusters can enhance their ability to travel upstream and facilitate targeted delivery of high doses of therapeutic agents^5^. On the other hand, in smaller capillaries, microrobots should ideally disperse into individual entities to allow unimpeded travel, with it being critical that they do not form irreversible clusters that can cause microvascular obstruction. Thus, lower magnetic interaction forces but higher magnetic anisotropy is likely to be favoured in such a situation. Moreover, additional design criteria should be met for biomedical microrobots, including the use of biocompatible materials. Surface functionalization with specific ligands will enhance cellular targeting and tailored sizes and shapes may facilitate cellular uptake^31,32^. Our fabrication pipeline allows for the control of multiple properties, including size, overall structure, and internal magnetic supradomain. Moreover, various therapeutic can be encapsulated into the microrobot to work as a drug delivery system. After polymerization, functionalization can be implemented, unlocking the potential of these microrobots for diverse biomedical applications.

## Methods

### Fabrication of soft microrobots

Polyethylene glycol diacrylate (455008, Sigma-Aldrich, USA) and a water-based ferrofluid (EMG-700, Ferrotec, USA) with a stock magnetic nanoparticle concentration of 5.8% vol (Fig. S8) and lithium phenyl (2,4,6-trimethyl) phosphinate (LAP) (900889, Sigma Aldrich, USA) were mixed with Dulbecco’s phosphate-buffered saline (DPBS) under ultrasound sonication and vortex mixing to prevent the formation of MNP aggregates. The resulting suspension was delivered to a flow-focusing geometry within a droplet-based microfluidic device to generate precursor droplets.

Photolithography masks containing the microfluidic channel pattern were printed onto a high-resolution film photomask (Micro Lithography Services, UK). Master moulds were created by spin coating with a 40 μm-thick layer of SU-8 photoresist (GM1070, MicroChem, USA). After pre-baking, UV lithography, development, and post-baking, the resulting structure was used as a master for polydimethylsiloxane (PDMS) moulding. Specifically, a 5 mm-thick layer of PDMS (Sylgard 184 A: B, Dow Corning, USA) was poured onto the master wafer and cured at 70 °C overnight. After curing, the fluidic layer was peeled off the mould and diced into individual devices using a laboratory scalpel. Inlet and outlet ports were created at appropriate locations using a hole-puncher (Technical Innovations, USA). The structured PDMS layer was bonded to a 24 × 75 mm glass slide (Epredia, USA) coated with a thin layer of PDMS after oxygen plasma treatment.

Hydrogel droplets were generated using the fabricated devices. Devices contains three inlets: one to introduce the HFE-7500 oil carrier phase (3 M, USA) with 1.25% v/v surfactant (RAN Biotechnologies, USA) and two to introduce the PEGDA-MNP hydrogel. Three LineUp Flow EZ pressure pumps (Fluigent, France) were used to control volumetric flow rates of the input streams, and droplet visualization was performed using an inverted Ti-E microscope (TI-DH, Nikon, Japan) equipped with a high-speed camera (Motion Pro Y5.1, Niederoenz, Switzerland). After their production, precursor droplets were transferred to a 500 μL Eppendorf tube for storage at 4 °C.

On-chip photopolymerization of precursor droplets was achieved through continuous LED irradiation at 365 nm (LEDMOD365. 1050.V2, Omicron, Germany) and illumination powers up to 1000 mW·cm^−2^. For off-chip photopolymerization, the same LED was integrated with a motorized Halbach cylinder. Precursor droplets collected from the droplet-based microfluidic were exposed to magnetic actuation (either UMF or RMF) for 30 s before a 5-min exposure to UV radiation (1000 mW·cm^−2^). The soft microrobot products are stored in HFE-7500 oil at 4 °C prior to use.

Achievable sizes: microrobot size can be controlled either by varying nozzle geometry or applied volumetric flow rates. A lower size limit on the formed droplets exists due to the inhibitory effect of oxygen on the photopolymerization of PEGDA^33^. To overcome this limitation, both for on-chip gelation and off-chip gelation, we implemented nitrogen protection to reduce oxygen content within droplets, enabling continuous photopolymerization of hydrogel droplets with diameters below 20 μm.

Achievable shapes: Below a critical concentration of 20 % (*v*/*v*) PEGDA, insufficient monomer is present, preventing chain-growth polymerization^34^. In contrast, with an excessive PEGDA concentration of 26 % (*v*/*v*), flocculation of MNPs is observed since PEG molecules act as a depletant that is preferentially excluded from the vicinity of the MNPs. This leads to an attractive depletion force between the MNPs and the formation of aggregates, which eventually causes blockage of the microfluidic channel. Between these extremes, spherical, doublet, and ellipsoidal structures can be generated. It should be noted that at elevated PEGDA fractions the shape morphs from a doublet to an ellipsoid due to a higher crosslinking rate. Additionally, it is important to remember that the concentration of PEGDA plays an important role in the droplet generation process. For example, as the concentration of PEGDA increases, the viscosity of the precursor solution will also increase, necessitating higher shear forces to segment droplets. Excessively high concentrations of PEGDA will also lead to channel blockage. On-chip gelation produces ellipsoidal microrobots of tuneable aspect ratio without any magnetic stimuli, while off-chip gelation enables more convenient magnetic actuation before photopolymerization, permitting the design of anisotropic magnetic supra-domains. In addition to this, an important feature of off-chip gelation under an RMF is the ability to generate structural anisotropy. For example, a doublet structure can be observed at high PEGDA concentrations when photopolymerization occurs under an RMF. This can be explained by the arrested coalescence^13,35^ and fusion of magnetic precursor droplets during the crosslinking process, under dynamic interactions driven by the RMF. Here, droplets initially approach each other through dipolar interactions and fluidic shear forces, leading to the displacement of fluid between the droplets. Subsequently, van der Waals forces accelerate thinning of the interstitial film, resulting in the formation of a pore that connects the droplets. Since the pore is unstable and expands, droplets eventually fuse.

Achievable supra-domains: Before the polymerization of precursor droplet, MNPs are able to freely move inside a droplet, with the motion of MNPs being influenced by the drag forces acting on them. As they move through the encapsulated fluid, they induce secondary fluid flows and create hydrodynamic force on neighbouring MNPs. Such hydrodynamic interactions will also influence the alignment, aggregation, or dispersion of MNPs.

For a more detailed fabrication pipeline, please see Fig. S9.

### Motorized Halbach cylinder

This magnetization system comprised a hollow cylindrical permanent array (HCPMA) mounted on a stepper motor. In order to magnetize and manipulate MNPs inside the droplet prior to photopolymerization, the device provides a UMF of 400 mT and a uniform RMF at frequencies between 0 and 10 Hz. The motorized Halbach cylinder was constructed by mounting a layered support structure for magnets consisting of a laser-cut 6 mm acrylic sheet to the shaft of a brushed DC gearmotor (E192.24.5, Micro Motors, Italy). To ensure that steady state rotation requires as little torque as possible from the motor, the motor was mounted vertically, and the array rested upon a lubricated needle roller thrust bearing. Rotational frequencies, measured using an LED, photodiode, and piece of opaque tape, were found to vary linearly with an applied voltage up to 20 V (Fig. S10).

### Integrated imaging and magnetic manipulation system

The magnetic micromanipulation system (MFG-100-i, MagnebotiX, Switzerland) consists of eight electromagnets arranged in a single hemisphere, integrated into an inverted confocal microscope (Nikon Eclipse Ti2 Yokogawa CSU-W1 unit with a Hamamatsu C13440-20CU Digital CMOS camera). The setup provides a working distance of 2 cm, making it compatible for use with microfluidic devices and imaging chambers (e.g., Fast-read@102, Biosigma, Italy). Sample was added to the imaging chamber and positioned between the objective lens and the magnetic manipulation system (Fig. S11). The system is able to generate magnetic fields with magnitudes up to 50 mT and operates at a maximum frequency of 2 kHz.

### VSM measurements

The magnetic properties of soft microrobots were measured by vibrating sample magnetometry (EZ-VSM, Mircosense). We conducted tests on three types of soft microrobots, each featuring distinct magnetic supra-domains and MNP concentrations. (Fig. S12).

### Hydrodynamic and magnetic simulations of the MNP assembly process within droplets and the collective behaviours of magnetic microrobots

In the simulation of the MNP assembly process within a precursor droplet, the superparamagnetic nanoparticle is simplified as spherical particle (radius ${R}_{p}$) with a magnetic dipole at the center of mass. The dipole direction is always aligned with the applied external magnetic field. To simulate structure formation in a droplet confinement, the interface is represented as a solid spherical shell (radius *R*). We carried out simulations with a given confinement ratio ($R/{Rp}$) of 20. 1000 MNPs are used and randomly initialized inside the shell. When a UMF is applied, the dipolar interactions give rise to “tail-to-head” connections of the MNPs along the direction of the external field, leading to the formation of multiple chains. Under an RMF, the attractive interactions between rotating dipoles aggregate the chains into planar disks, while their vertical repulsion finally results in stacks of parallel disks (Supplementary Video 2). Both chain and disk structures give rise to a patterned and aligned distribution of MNPs inside of the microrobot, leading to magnetic shape and crystallographic anisotropy. To characterize the distribution of the MNPs, we define an isotropy order *γ* = < |*z*_*p*_| > /< |*y*_*p*_| >  where < |*z*_*p*_| > and < |*y*_*p*_| > are the averaged absolute displacement of MNPs relative to the droplet center. The extent of anisotropy can be tuned by adjusting the strength of the external magnetic field and varying the dimensionless parameter, $\beta$,which is defined as the ratio between the magnetic interaction force $(3{\mu }_{0}{m}^{2}/4\pi {{R}_{p}}^{4})$ and hydrodynamic force $(6\pi \mu \omega {{R}_{p}}^{2})$. Here ${\mu }_{0}$ is the magnetic permeability, $m$ is the magnitude of dipole moment, $\eta$ is the fluid viscosity$,$ ${and}$ $\omega$ is the angular rate of the RMF. We studied the evolution of the isotropic order $\gamma$ under increasing magnetic field strength from $\beta \approx 0.1$ to $\beta \approx 11.2$.

Lattice Boltzmann method. The open-source package ‘Ludwig’, a parallel code for the simulation of complex fluids^36^, was used to perform hydrodynamic simulations. Ludwig employs the lattice Boltzmann method (LBM) to solve Navier-Stokes equations. The LBM^37^ has been extensively used in computational fluid dynamics, but we briefly introduce aspects of the LBM for the reader’s convenience. In the LBM, on each lattice, the fluid is treated as a cluster of particles. They collide and stream in discrete directions with probabilities given by the distribution function ${f}_{i}\left(r,t\right),$ which is governed by the lattice Boltzmann equation (LBE)

1 $${f}_{i}\left(r+{c}_{i}\Delta t,t+\Delta t\right)-{f}_{i}\left(r,t\right)={\varOmega }_{i}$$

where ${\varOmega }_{i}$ is the collision operator. Additionally, the Bhatnagar-Gross-Krook (BGK) approximation is often adopted.

2 $${\varOmega }_{i}=-\frac{1}{\tau }\left[{f}_{i}\left(r,t\right)-{f}_{i}^{{eq}}\left(r,t\right)\right]$$

This assumes that the fluids locally relax to equilibrium over a characteristic time scale, $\tau$. Based on the Maxwell distribution, the equilibrium ${f}_{i}^{{eq}}$ is given as

3 $${f}_{i}^{{eq}}={\omega }_{i}\rho \left[1+\frac{1}{{c}_{s}^{2}}\left({c}_{i}\cdot u\right)+\frac{1}{2{c}_{s}^{4}}{\left({c}_{i}\cdot u\right)}^{2}-\frac{1}{2{c}_{s}^{2}}\left(u\cdot u\right)\right]$$

Here ${c}_{s}=\frac{1}{\surd 3}\frac{\Delta x}{\Delta t}$ is the speed of sound, $\rho$ is the fluid density and $u$ is the fluid velocity. The lattice velocities ${c}_{i}$ and weight factors ${\omega }_{i}$ are given by the lattice vectors model. The so-called D3Q19 model is used in this work.

For small Mach and Knudsen numbers, it has been shown, through the Chapman-Enskog expansion, that the lattice Boltzmann equation recovers the incompressible Navier-Stokes equations, i.e.

4 $$\nabla \cdot u=0$$

5 $$\rho \left(\frac{\partial u}{\partial t}+u\cdot \nabla u\right)=-\nabla p+\mu {\nabla }^{2}u$$

Using the distribution function, ${f}_{i}$, microscopic quantities can be calculated, such as the fluid velocity $u=\frac{1}{\rho }{\varSigma }_{i}{c}_{i}{f}_{i}$, pressure, $p={c}_{s}^{2}\rho$ and viscosity of the fluid $\mu =\frac{1}{2}\rho {c}_{s}^{2}\Delta t \, \left(2\tau -1\right)$.

The LBM has been shown efficient in computing hydrodynamics and is simple to parallelize. Accordingly, large-scale direct simulations become viable and allow detailed hydrodynamic interactions within reasonably large and experimentally relevant systems to be studied.

To account for the no-slip boundary condition on a moving particle surface, the ‘bounce back on links’ algorithm is applied^38^. Specifically, we consider a density-matched suspension ($\rho ={\rho }_{{fluid}}={\rho }_{{particle}}=1$) and set the LBM lattice spacing, ∆*x*$,$ to be 1 and time unit, ∆*t, to be* 1. Microrobots are modelled as spherical particles with a radius, *R* = 4.1∆*x*. To avoid overlap between solid boundaries, we employ a short-range repulsive potential when surface distance *d* < 0.1∆*x* ^39^.

Magnetic dipole-dipole interaction. The magnetic dipole-dipole interaction has been included in the Ludwig code^40,41^. A uniform magnetic field, *B*, can be applied to align dipoles. For simulating the collective motion of microrobots, a flat wall perpendicular to the rotating axis is used as the boundary condition, while the other boundaries remain periodic.

For the quantitative comparison between simulation and experiment are presented in Fig. S13.

Heterogenous swarm control. To conduct our simulations, we employed a doublet microrobot consisting of two spheres that are bonded together. It should be noted that our simulations encompassed a total of 600 spheres and each individual spherical microrobot comprises just a single sphere Computational parameters were set to ${L}_{z}=10R$ and ${L}_{x}={L}_{y}=80R$, with $R$ being the radius of the spherical microrobot. As part of an analysis of the assembly process and locomotion of formed “microrobots rafts”, we were interested in the area fraction occupied by microrobots. Accordingly, with 600 microrobots the overall area fraction is $\frac{600\pi {R}^{2}}{{L}_{x}{L}_{y}}\approx 29.5 \%$ as a starting point. The initial positions of the microrobots are analogous to the randomly distributed experiment. The local area fraction is measured by using a $7R\times 7R$ subdomain to scan the regions that contain microrobots.

### Particle-based magnetization simulations

Four thousand distributed and uniform particles of high relative magnetic permeability were utilized as surrogates for superparamagnetic MNPs, with each particle being approximated as a magnetic point dipole. The resulting magnetic dipole moment of each particle exposed to an external magnetic field was then used to calculate the forces and torques experienced by each microrobot, including their dipole-dipole interactions. As a magnetic stimulus to drive motion, a homogeneous RMF was chosen.

For any randomly sampled MNP $i$ in microrobots, its position and magnetic dipole are denoted as ${r}_{i}$ and ${m}_{i}$ respectially. The magnetic field ${H}^{{\prime} }$ generated by such MNP at position $r$ can be given as

6 $${H}^{{\prime} }=\frac{3{m}_{i}\cdot \hat{r}\hat{r}-{m}_{i}}{4\pi {\mu }_{0}{r}^{3}}$$

where ${\mu }_{0}$ is the constant vacuum permeability. $r$ and $\hat{r}$ are the length and direction of $r$, respectively.

When polarized by an external magnetic field, $H$, the magnetic dipole moment of particle $i$ is given as

7 $${{{{{{\boldsymbol{m}}}}}}}_{i}=4\pi {\mu }_{0}{a}^{3}\frac{{\mu }_{r}-1}{{\mu }_{r}+2}\left({{{{{\boldsymbol{H}}}}}}+\mathop{\sum }\limits_{j\ne i}\frac{3{{{{{{\boldsymbol{m}}}}}}}_{j}\cdot {\hat{{{{{{\boldsymbol{r}}}}}}}}_{{ij}}{\hat{{{{{{\boldsymbol{r}}}}}}}}_{{ij}}-{{{{{{\boldsymbol{m}}}}}}}_{j}}{4\pi {\mu }_{0}{r}_{{ij}}^{3}}\right)$$

where $a$ is the radius of the particle, and *μ*_*r*_ (= 1000) represents the relative permeability of the superparamagnetic material. We define ${r}_{{ij}}$ = ${r}_{i}-{r}_{j}$, and symbolize its length and direction using ${r}_{{ij}}$ and $\hat{{r}_{{ij}}}$ respectively. The second term within the parentheses represents the cumulative effect of all remaining particles within the system.

The calculation of the mutual interaction between MNPs necessitates the simultaneous determination of the magnetic dipole moments of all particles within the system. By relocating all unknown magnetic dipole moment terms from the right-hand side to the left-hand side of the equation, we arrive at the following equation for the determination of the magnetic dipole moments of all particles:

8 $$\frac{{{{{{{\boldsymbol{m}}}}}}}_{i}}{4\pi {\mu }_{0}{a}^{3}}-\frac{{\mu }_{r}-1}{{\mu }_{r}+2}\mathop{\sum }\limits_{j\ne i}\left(3{\hat{{{{{{\boldsymbol{r}}}}}}}}_{{ij}}{\hat{{{{{{\boldsymbol{r}}}}}}}}_{{ij}}-{{{{{{\boldsymbol{I}}}}}}}_{3}\right)\frac{{a}^{3}}{{{{{{{\boldsymbol{r}}}}}}}_{{ij}}^{3}}\frac{{m}_{j}}{4\pi {\mu }_{0}{a}_{{ij}}^{3}}=\frac{{\mu }_{r}-1}{{\mu }_{r}+2}{{{{{\boldsymbol{H}}}}}}$$

where ${I}_{3}$ denotes the 3 × 3 identity matrix. In practical implementation, the linear equation is resolved using the conjugate gradient method.

After obtaining the magnetic dipole moments of each MNP in the system, we are able to determine the forces and torques experienced by a microrobot. Given the insignificant relaxation time of magnetization, it is reasonable to assume that the magnetic dipole moment of each MNP remains consistently aligned with the direction of the magnetic field it encounters. Based on the principle of virtual work, the magnetic force between any two MNPs in the system can be explicitly formulated as:

9 $${F}_{{ij}}= 	\,{m}_{i}\cdot \nabla {H}_{{ij}}=\frac{3}{4\pi {\mu }_{0}{r}_{{ij}}^{4}}\left[\left({m}_{i}\cdot {m}_{j}\right){\hat{r}}_{{ij}}+\left({m}_{i}\cdot {\hat{r}}_{{ij}}\right){m}_{j} \right. \\ 	+\left. \left({m}_{i}\cdot {\hat{r}}_{{ij}}\right){m}_{i}-5\left({m}_{i}\cdot {\hat{r}}_{{ij}}\right)\right]$$

in which ${m}_{i}$ is the strength of the magnetic dipole moment of the *i*-th MNP, ${H}_{{ij}}$ denotes the magnetic field generated by the *j*-th MNP at the *i*-th MNP. ${\hat{r}}_{{ij}}$ represents the normalized direction from the *j*-th MNP to the *i*-th MNP.

The overall magnetic force experienced by each MNP results from interactions with all the other MNPs in the system. By summing up the interaction’s forces from all the enclosed MNPs, we can obtain an approximation of the force experienced by each microrobot. As a result, the rotational torque exerted on each microrobot can be determined by summing the torques acting on all the embedded MNPs as follows:

10 $${{{{{{\boldsymbol{\tau }}}}}}}_{1}={\sum }_{i}\left({{{{{{\boldsymbol{r}}}}}}}_{i}^{{\prime} }\times \mathop{\sum }\limits_{j}{{{{{{\boldsymbol{F}}}}}}}_{{ij}}\right)$$

Here, the index *j* iterates over all the MNPs in the system, while index *i* iterates over the MNPs within the microrobot; and ${r}_{i}^{{\prime} }$ denotes the position of the *i*-th MNP relative to the center of the microrobot. The revolution torque of the microrobot can be analogically derived as

11 $${{{{{{\boldsymbol{\tau }}}}}}}_{2}={{{{{{\boldsymbol{r}}}}}}}_{C}\times \mathop{\sum }\limits_{i}\mathop{\sum }\limits_{j}{{{{{{\boldsymbol{F}}}}}}}_{{ij}}$$

where ${r}_{C}$ indicates displacement of the microrobot relative to the center of mass of the system. In that case, the dipolar magnetic force, ${F}_{r},$ is the projection of the overall interaction force perceived by each microrobot along the direction ${\hat{r}}_{C}$, i.e.

12 $${{{{{{\boldsymbol{F}}}}}}}_{r}={\hat{{{{{{\boldsymbol{r}}}}}}}}_{C}\cdot \mathop{\sum }\limits_{i}\mathop{\sum }\limits_{j}{{{{{{\boldsymbol{F}}}}}}}_{{ij}}$$

### Supplementary information

Peer Review File

Supplementary information

Supplementary Video 1

Supplementary Video 2

Supplementary Video 3

Supplementary Video 4

Supplementary Video 5

Supplementary Video 6

Supplementary Video 7

Supplementary Video 8

## Data Availability

More data which relevant to fabrication and control experiment are available from S.S (simone.schuerle@hest.ethz.ch) on reasonable request. Source data are provided with this paper.

## Code availability

The code for hydrodynamic simulation of magnetic microrobots use in current study are publicly available on GitHub (https://github.com/ludwig-cf/ludwig). The code for particle-based magnetization simulation is available as a Python package on GitHub (https://github.com/ZZzyc001/MagneticLinePlotting).
