# Supplementary material for: Programming structural and magnetic anisotropy for tailored interaction and control of soft microrobots
Source: Commun Eng. 2024 Jan 5;3:7. doi: 10.1038/s44172-023-00145-5 (PMC10955931; doi:10.1038/s44172-023-00145-5)
Supplement: Supplementary file 2 — Supplementary information [file 44172_2023_145_MOESM2_ESM.docx]

Supplementary material

**Programming structural and magnetic anisotropy for tailored interaction and control of soft microrobots**

Yimo Yan†^1^, Chao Song†^2^, Zaiyi Shen^3^, Yuechen Zhu^4^, Xingyu Ni^4^, Bin Wang^5^, Michael G. Christiansen^1^, Stavros Stavrakis^2^, Juho S Lintuvuori^6^, Baoquan Chen^4^, Andrew deMello^2^*, Simone Schürle^1^*

***Corresponding authors**

**E-mail address:**

**Simone Schürle:**[**simone.schuerle@hest.ethz.ch**](mailto:simone.schuerle@hest.ethz.ch)

**Andrew deMello: andrew.demello@chem.ethz.ch**

**Affiliations:**

1. Department of Health Science and Technology, Institute for Translational Medicine, ETH Zürich, Switzerland
2. Department of Chemistry and Applied Biosciences, Institute for Chemical and Bioengineering, ETH Zürich, Switzerland
3. Department of Mechanics and Engineering Science, College of Engineering, Peking University, China
4. National Key Laboratory of General Artificial Intelligence, Peking University, China
5. National Key Laboratory of General Artificial Intelligence, BIGAI, China
6. CNRS, LOMA, University of Bordeaux, France

**
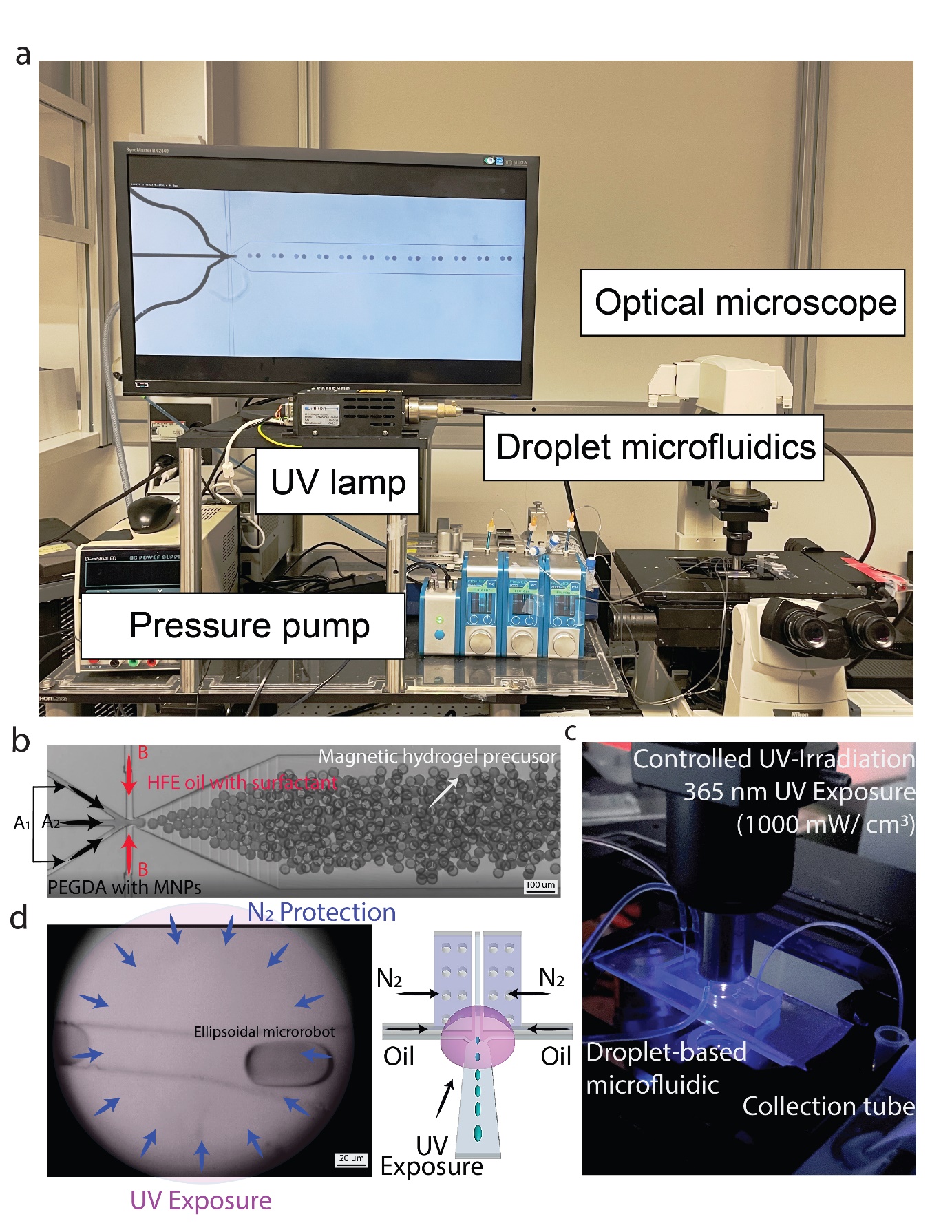
Fig. S1.** **a** Set-up of the droplet-based microfluidics system. **b** Generation of magnetic precursor droplets in microfluidic channels. A discrete phase of PEGDA solution (A_1_ and A_2_) and a continuous phase of HFE oil (B). The two inlets for PEGDA serve as a backup for each other, reducing the risk of channel clogging. **c** Set-up of the on-chip gelation system. **d** On-chip gelation process involves the focused exposure of UV irradiation in the junction area of droplet-based microfluidic channels using an optical lens while ensuring a surrounding area of nitrogen protection. This approach enables the triggering of photopolymerization, whilst also allowing the resulting structures to maintain the desired aspect ratio.


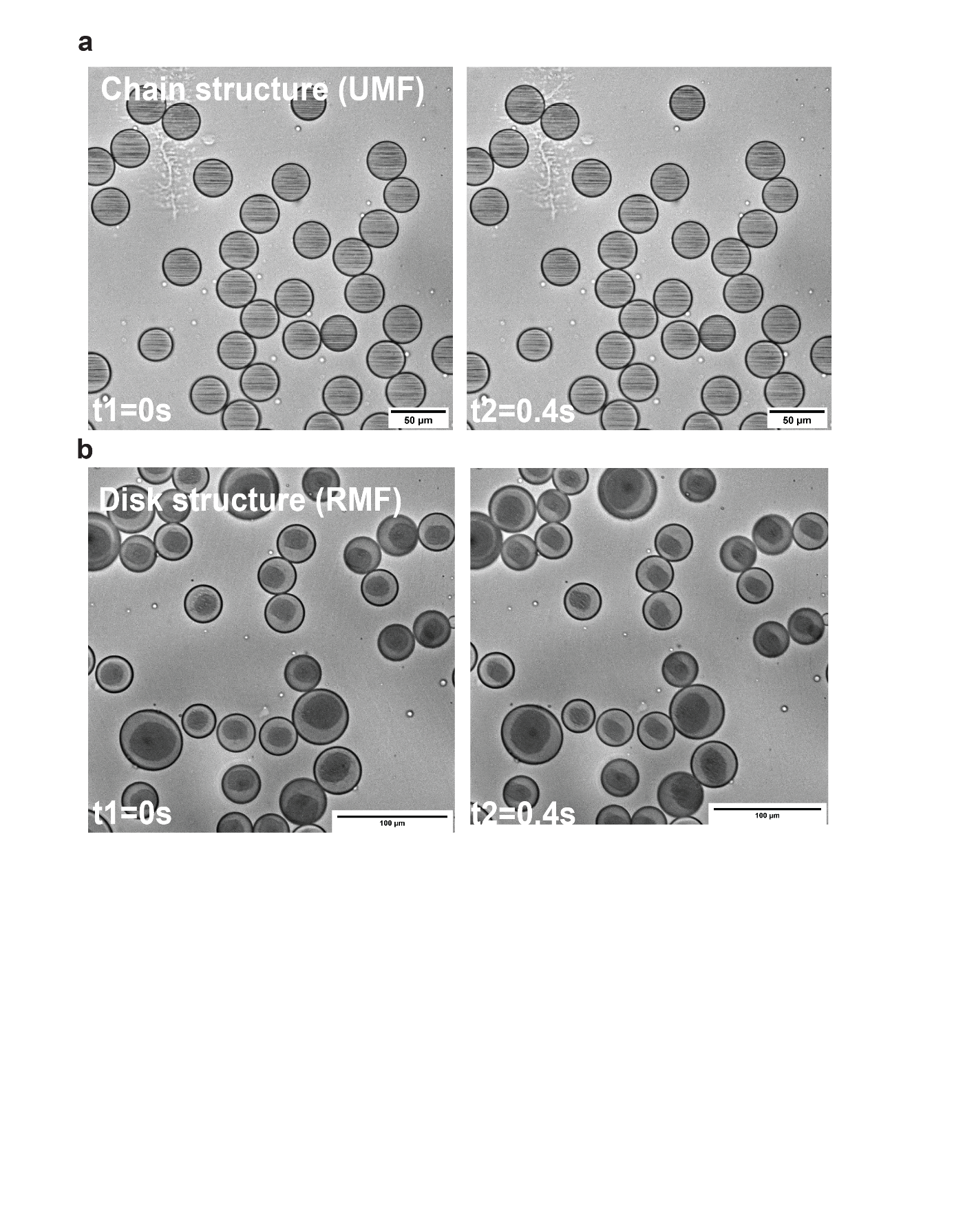


**Fig. S2. a** MNPs assembly process within magnetic precursor droplets under UMF. Chain structures can be achieved and observed. **b** MNPs assembly process within magnetic precursor droplets under RMF. Disk structures can be achieved and observed.


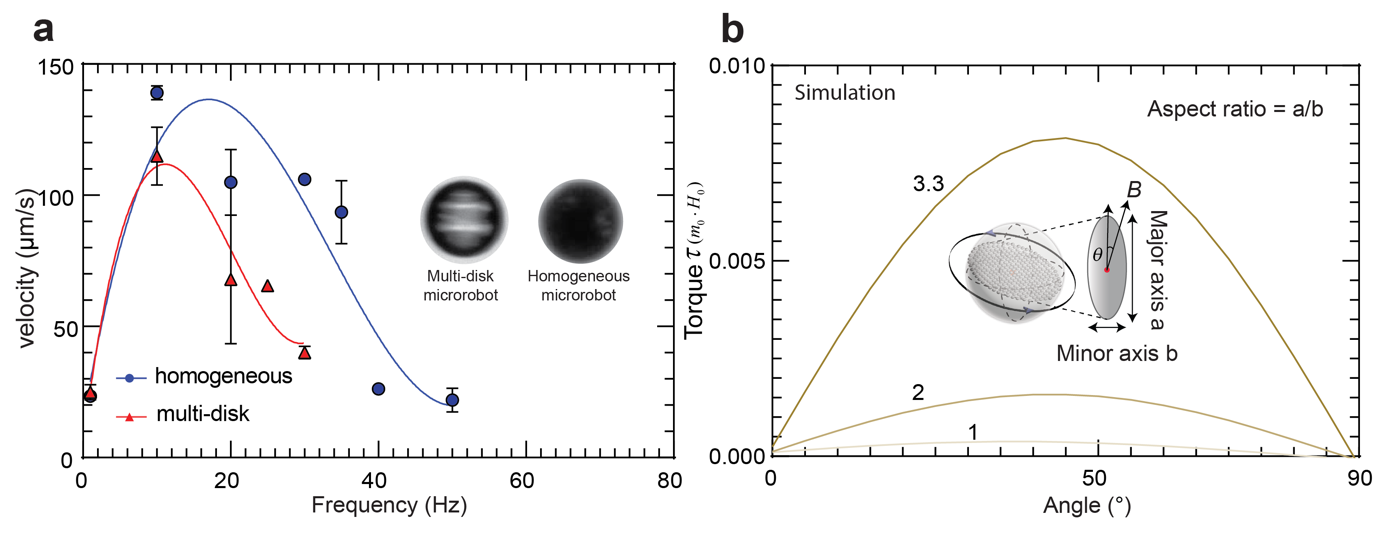


**Fig. S3.** **a** Torque-driven locomotion of microrobots is dependent on their magnetic anisotropy. The translational velocity of the microrobot (having identical diameters of 25 µm) under a constant RMF increases with increasing magnetic anisotropy. The multi-bundle microrobot, with its distinct magnetic anisotropy, exhibits a higher step-out frequency than the homogeneous microrobot. Error bars represent the SD of the mean. **b** Impact of internal magnetic supradomain aspect ratio on the torque of microrobot induced by an RMF. The simulation results indicate a positive correlation between the aspect ratio and the induced torque, which ultimately leads to a higher step-out frequency.


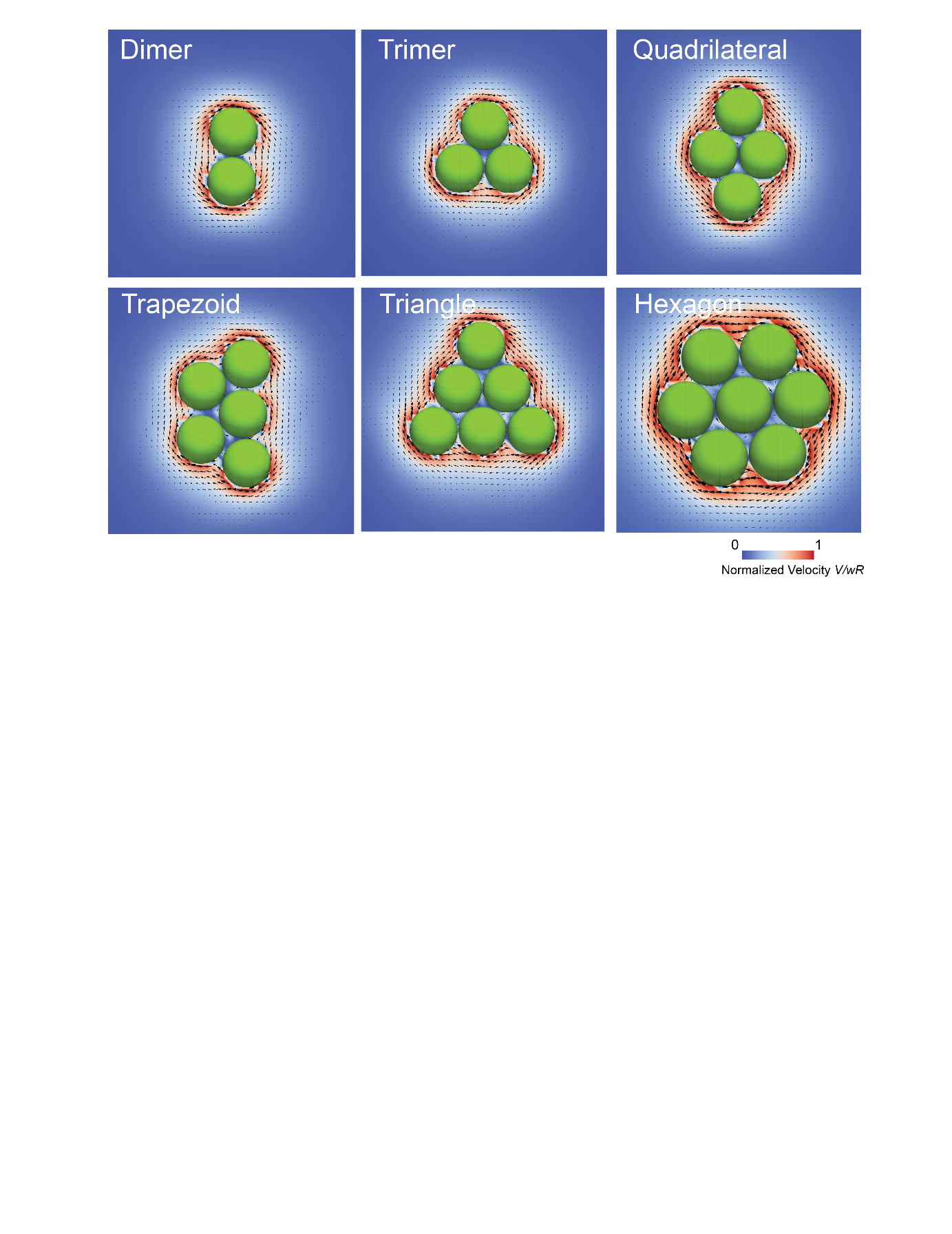


**Fig. S4.** Hydrodynamic simulation of multi-disk microrobot clusters in dynamic steady state under an RMF.


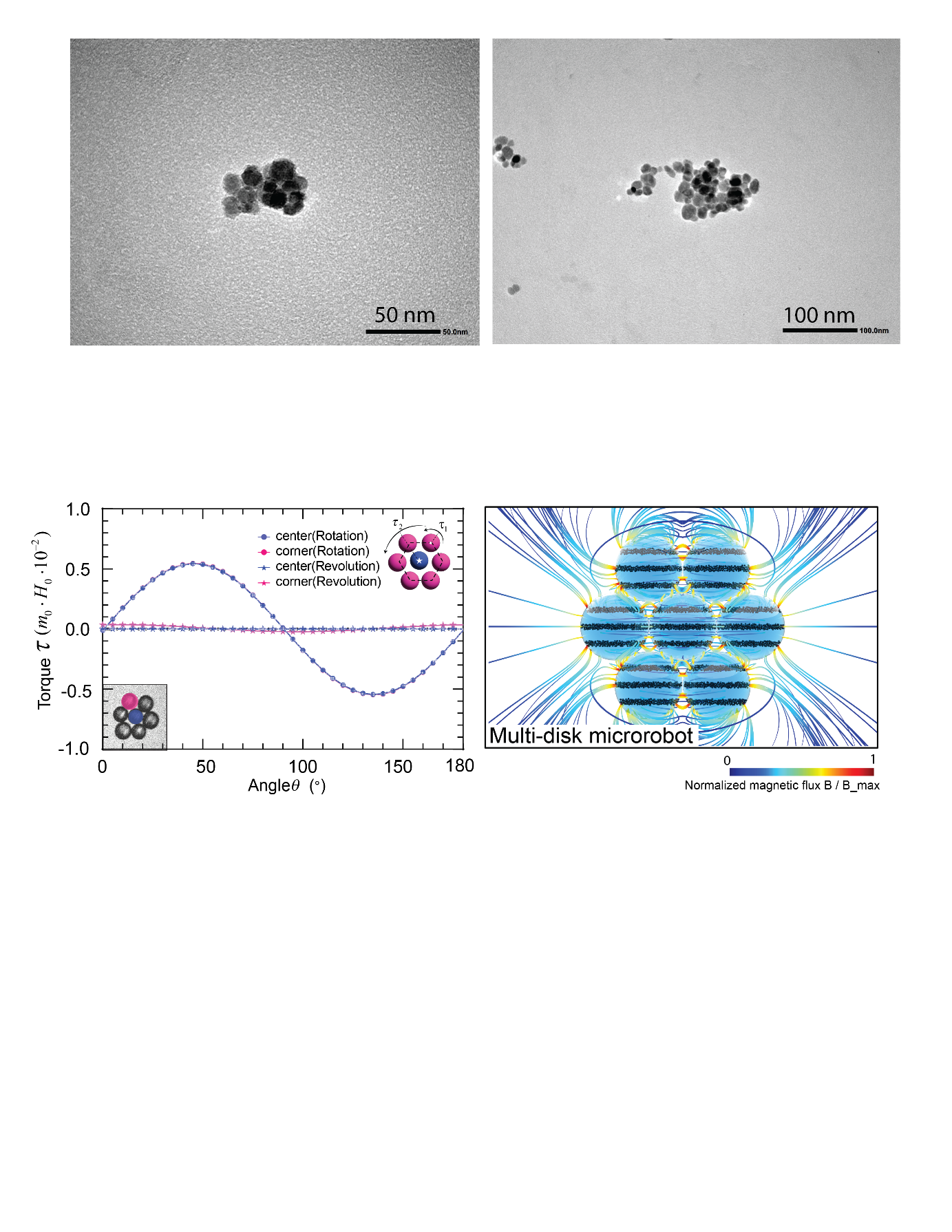


**Fig. S5.** The simulated magnetic flux of a hexagonally ordered structure of homogenous and multi-disk microrobots under an RMF. The resulting magnetic torque leading to rotation and revolution of center and corner microrobots respectively is analysed and quantified.

**
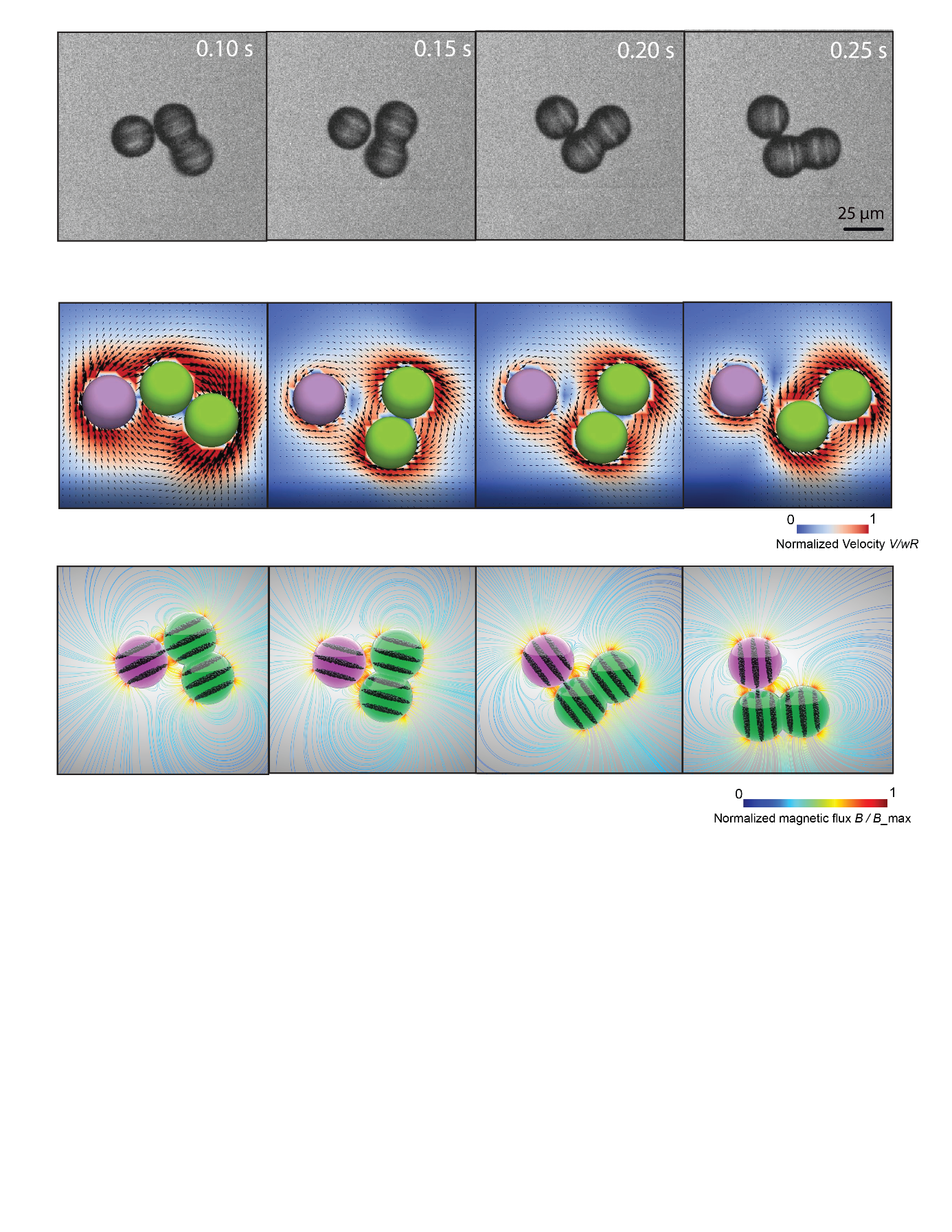
**

**Fig. S6.** Snapshots and simulation of the magnetization and hydrodynamics of microrobot pairs comprising doublet and spherical microrobots.


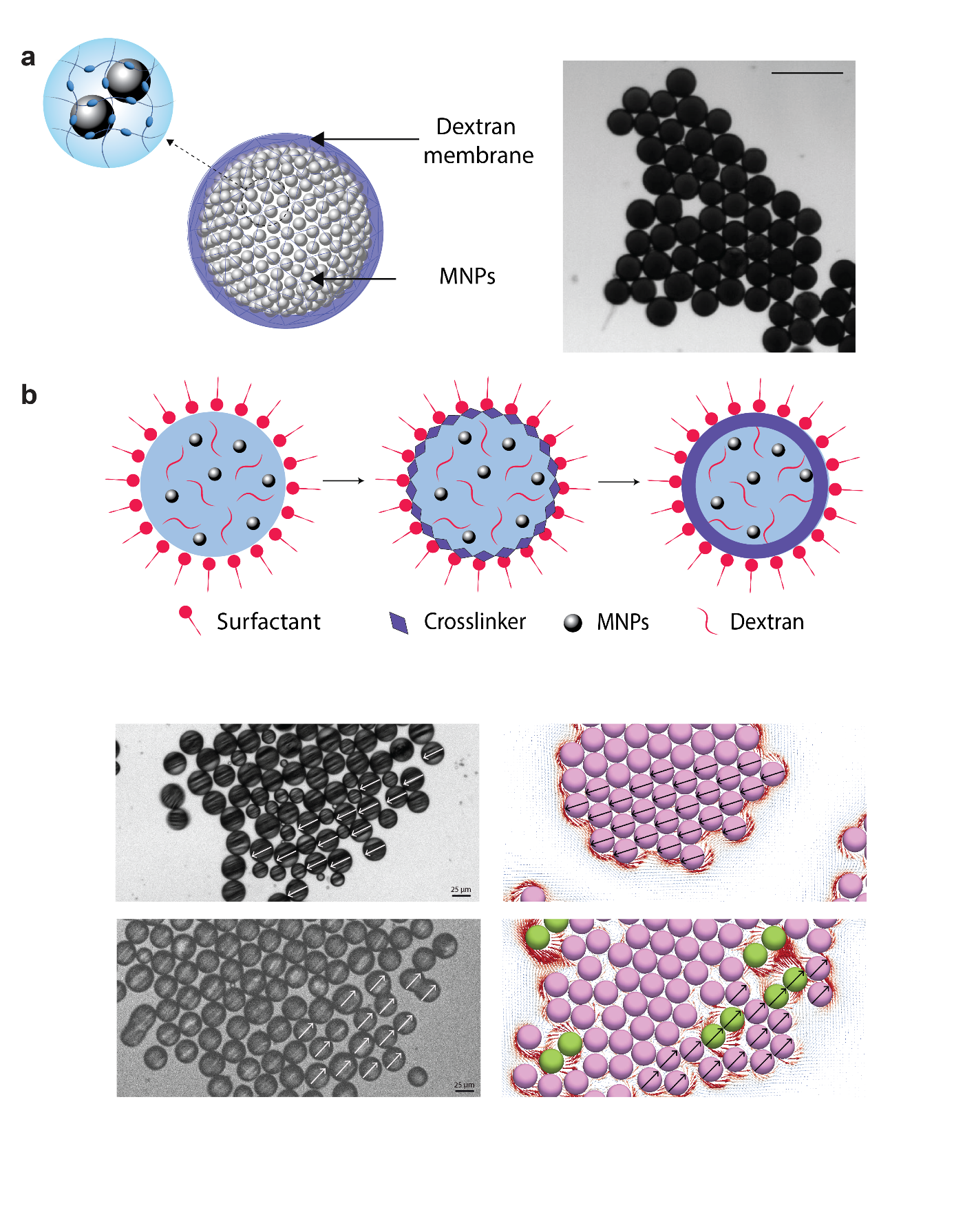


**Fig. S7.** **Visualization of individual microrobot orientation under an RMF.** Experimental images of a microrobot swarm composed of different entities and driven by an RMF at 2 Hz. The simulated flow and magnetic field during the assembly process are shown. Arrows indicate the direction of rotation, which aligns with the external magnetic field.


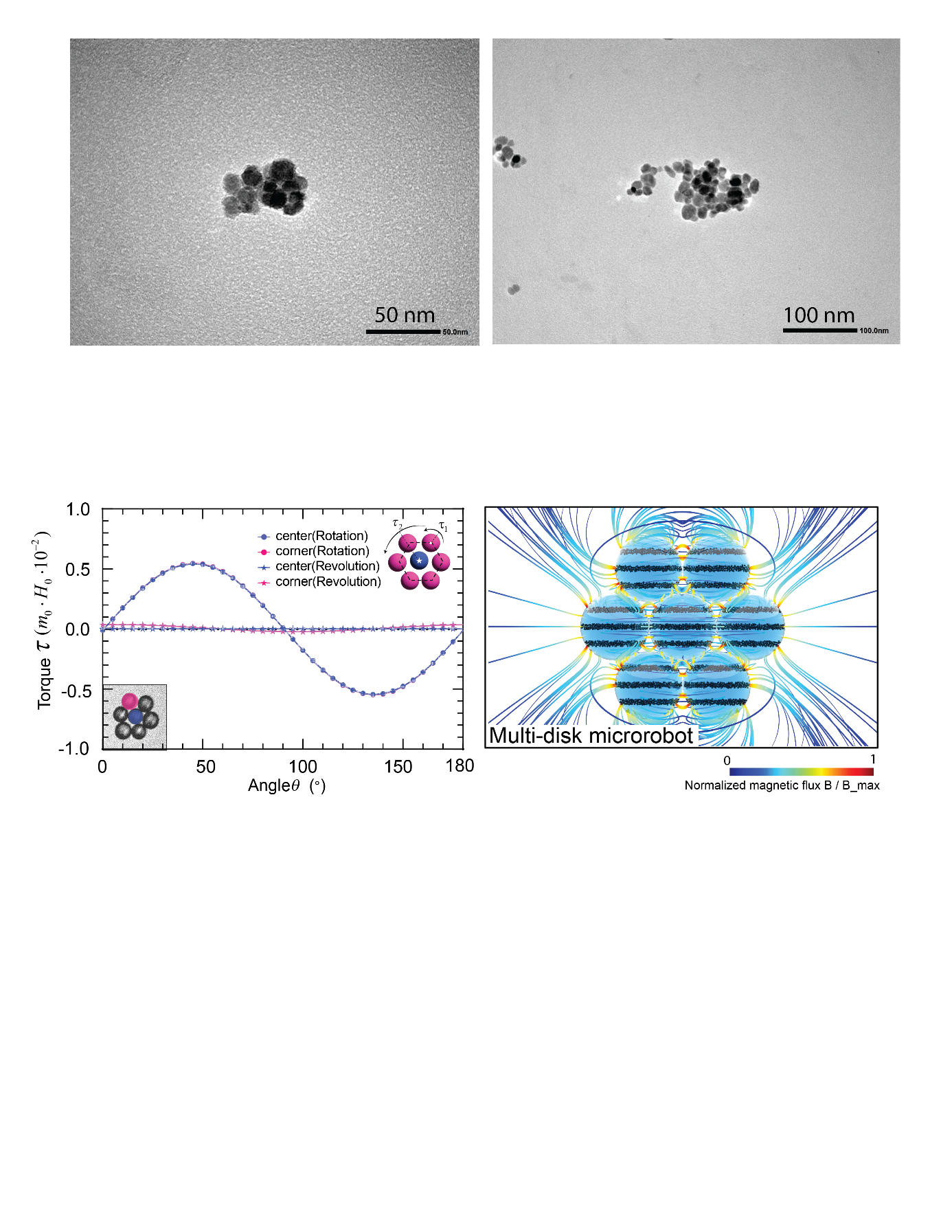


**Fig. S8.** Transmission electron microscopy (TEM) image of the employed commercial magnetic nanoparticles (EMG-700, Ferrotec).


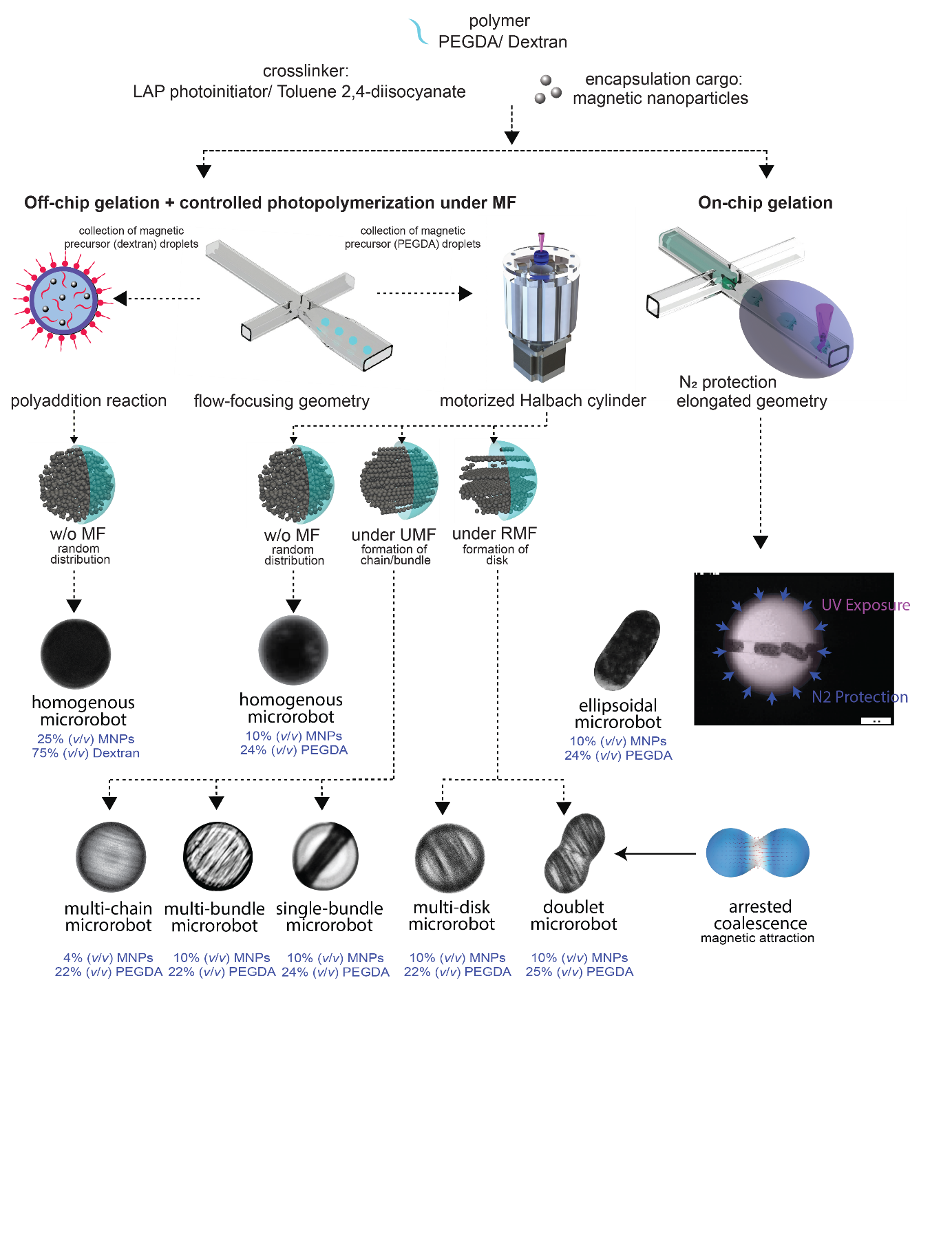


**Fig. S9 Fabrication scheme to achieve structures containing tailored magnetic supradomains and distinct shapes.** The process involves a combination of off-chip gelation, controlled photopolymerization under magnetic fields, which results in the production of homogenous^1^, multi-chain, multi-bundle, and multi-disk as well as doublet microrobots. By implementing on-chip gelation, a variety of aspect ratios for ellipsoidal microrobots can be achieved. Higher concentration of MNPs in a homogenous microrobot can be achieved by implementing off-chip crosslinking of the dextran precursor droplets.


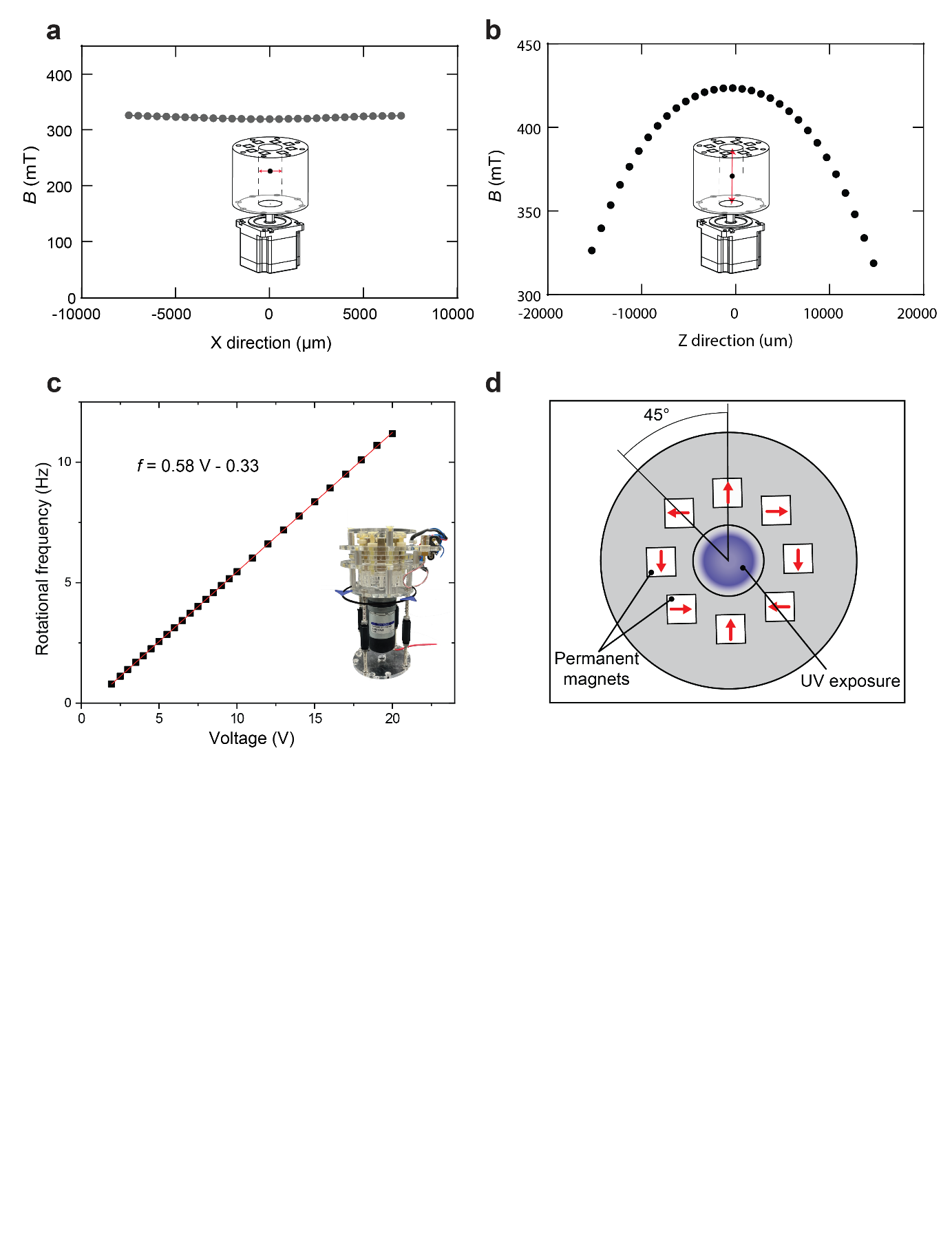


**Fig. S10.** **Characterization of the motorized Halbach cylinder. a** Variation of magnetic field strength along the x-axis. **b** Variation of magnetic field strength along the y-axis. **c** Relationship between the frequency of the RMF and the input voltage. **d** Configuration of permanent magnets in the motorized Halbach cylinder device.

**
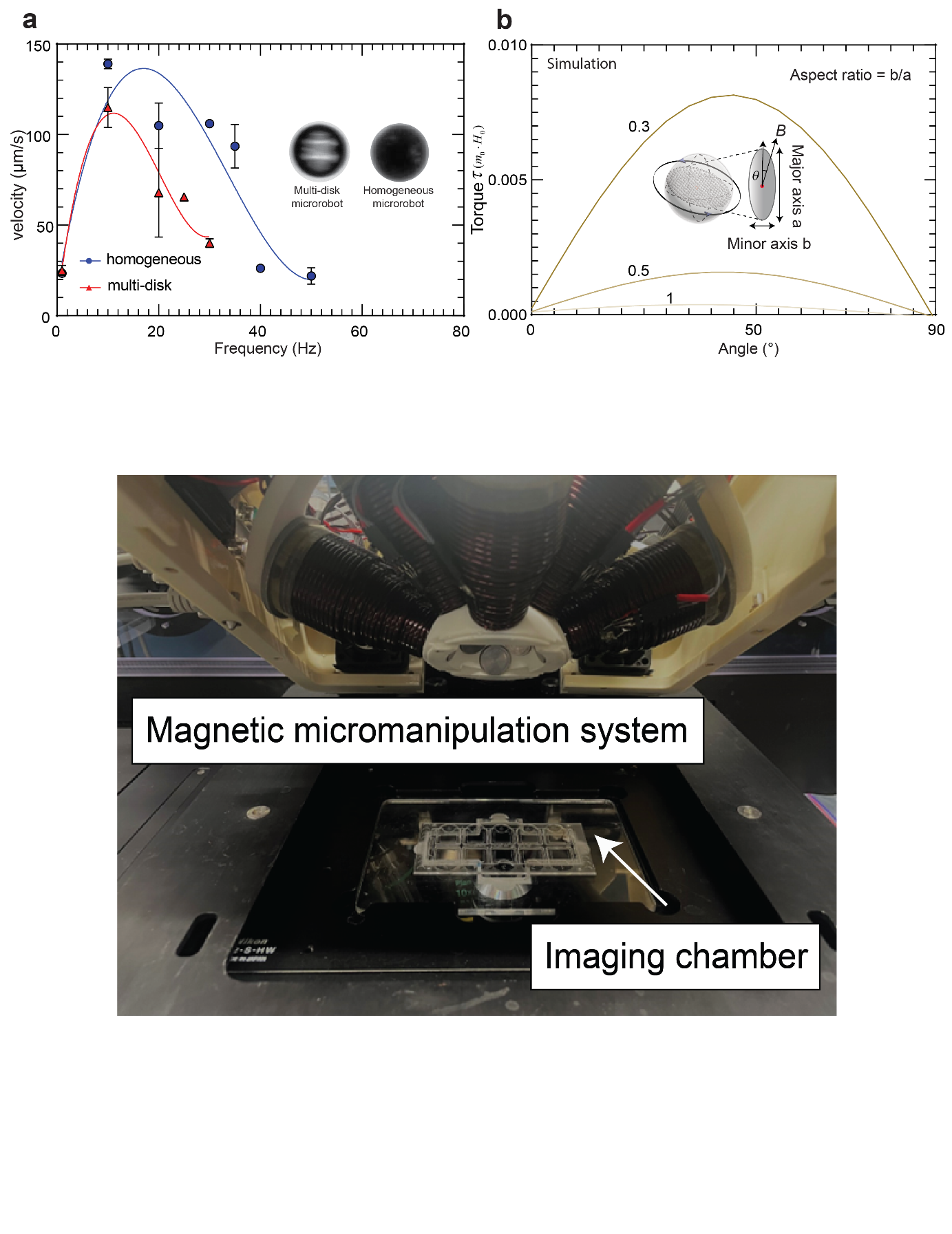
**

**Fig. S11.** Experimental setup of magnetic micromanipulation system and imaging chamber.

**
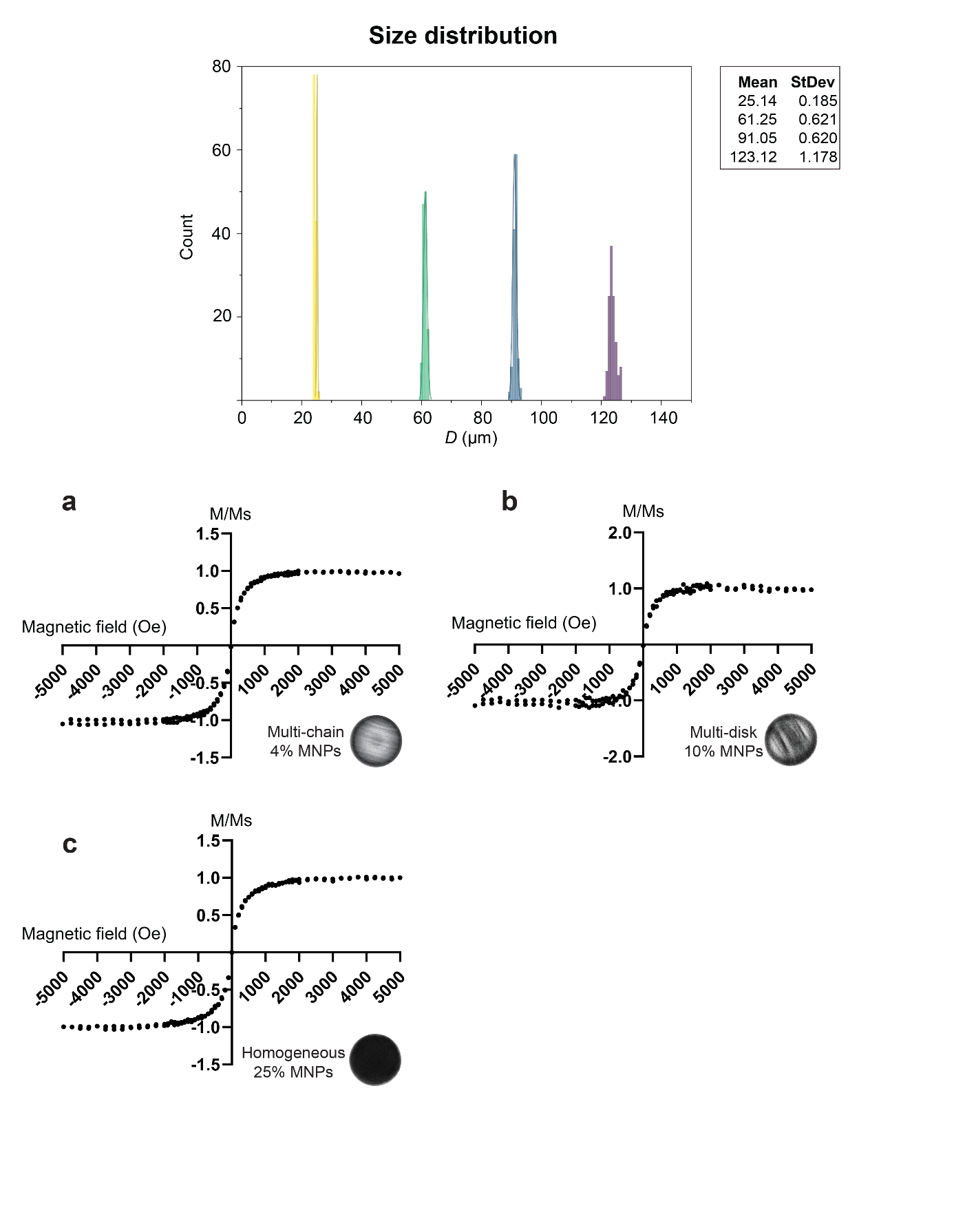
**

**Fig. S12.** **a** M/Ms vs. H curve for multi-chain microrobots that contain 4% (v/v) MNPs obtained by VSM. **b** M/Ms vs. H curve for multi-disk microrobots that contain 10% (v/v) MNPs obtained by VSM. **c** M/Ms vs. H curve for homogenous microrobots which contain 25% (v/v) MNPs obtained by VSM.

**Supplementary Notes 1**

To quantitatively compare hydrodynamic simulation and experiment, we calibrate the dimensionless parameter $\beta=\frac{\frac{3\mu_{0}m^{2}}{4\pi R_{p}^{4}}}{6\pi\mu\omega R_{p}^{2}}=\frac{\mu_{0}m^{2}}{8\pi^{2}\mu\omega R_{p}^{6}}$ which is defined as the ratio between the typical magnetic interaction force and the hydrodynamic interaction force.^2^ First, we obtain $\beta$ in experiment by measuring the separation rate when two touching microrobots on a surface are subjected to an external field (20 mT) perpendicular to the wall. The two microrobots separate due to the magnetic repulsion (Supplementary Fig.1a), which is given by $F_{m}=\frac{3\mu_{0}m^{2}}{4\pi r^{4}}$. This magnetic repulsion is balanced by fluid drag $F_{m}=F_{d}=6\xi\pi\mu R_{p}u_{s}$, where $u_{s}$ is the separating rate and $\xi$ is a coefficient due to the influence from the wall. So, the dimensionless parameter $\beta=\frac{\xi u_{s}r^{4}}{\omega}R_{p}^{5}$ is obtained. Faxén’s correction gives $\xi=1.8$. For the test case (Supplementary Fig.2a), we measured *u_s_* = 117 µm/s, separating distance *r* = 35.5 µm and the radius of the robot *R_p_* = 15µm. For quantitative comparison between hydrodynamic simulation and experiment, a rotating field (20 mT) of frequency $f=1 Hz$ is applied. These give $\beta=70$. We perform a simulation with $\beta=70$. By setting the same initial positions of the robots and the same initial external field orientation, we compare the trajectories and the interdistance between the two robots. The simulation results agree with experimental observation (Supplementary Fig.2b and 2c).


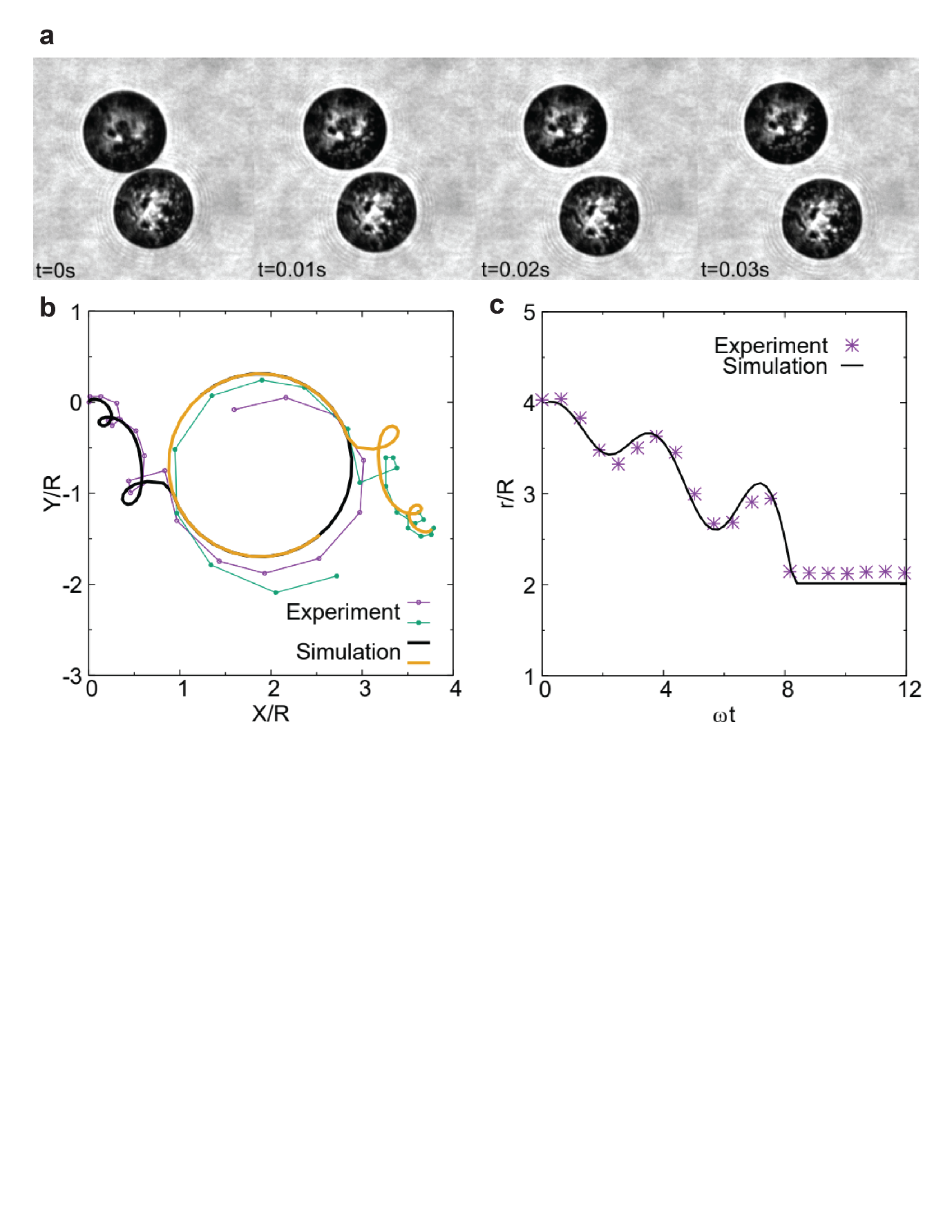


**Fig. S13. Quantitative comparison between simulation and experiments. a** Two touching microrobots settle on a flat surface. An external magnetic field perpendicular to surface is applied. The two robots separate due to magnetic repulsion. The separating rate is measured to estimate the magnetic moment of the microrobot. **b** Trajectories of two interacting rotating microrobots. **c** Time evolution of the interdistance between two rotating microrobots.

| **Strategy** | **Targeting** | **Advantage** | **Disadvantage** | **Ref.** |
| --- | --- | --- | --- | --- |
| Nanoparticle  (non-magnetic) | No | Flexibility, high stability in vivo, increase compound half-life, higher delivery efficacy | Inorganic nanoparticle may trigger the immune system | 1-5 |
| Liposome | No | Similarity with cell membrane, low system toxicity, high variety of drug encapsulation | Sensitivity to sterilization methods, low stability in circulation, low reproducibility in liposome loading and size control, short shelf-life | 6-10 |
| Extracellular vesicle (EV) | Can be modified to enhance tissue specificity | High biocompatibility,  low immunogenicity, functional complexity | Low efficiency of EVs isolation method, lack of clinical application | 11-14 |
| Polymeric microparticle  (non-magnetic) | No | Biocompatible and biodegradable, can achieve large scale production, able to both entrap hydrophilic and hydrophobic drugs, control release, protect drug from metabolic degradation, prolong residence time | Burst effect | 15-18 |
| Magnetic soft microrobot | High | Biocompatible and biodegradable, can achieve large scale production, able to both entrap hydrophilic and hydrophobic drugs, control release, protect drug from metabolic degradation, prolong residence time, high targeting ability under magnetic field control | Burst effect, lack of clinical application | This study |

**Table. S1.** Comparison of exiting drug delivery system for biomedical application.

| Technique type | Material | Advantage | Disadvantage | Ref. |
| --- | --- | --- | --- | --- |
| Two photon polymerization | GelMA hydrogel, magnetic nanoparticles | High precision, Biocompatible | Low throughput due to the device size constrains, complex fabrication procedure, costly, slow fabrication process | 19 |
| 3D direct laser writing and physical vapor deposition | SU8(Ni/Ti) | Arbitrary shape can be designed | Non-biocompatible, non-biodegradable, costly, slow fabrication process | 20 |
| Stereolithography (SLA) | Resign including photo-activated monomers, hybrid polymer, epoxides, DC 100 (high accuracy), DC 500, DL 350/360 (high flexibility), AB 001, GM 08 (high flexibility), DM 210, DM 220 | Easy to perform | Slow printing rate, limited choice of material, low precision | 21 |
| Sputtering | Ni, Au, commercial silica particles | High step out frequency | Non-biocompatible, non-biodegradable, complex fabrication procedure | 22 |
| Emulsion with follow-up magnetic programming | Magnetic nanoparticles, poly (ethylene glycol) diglycidyl ether (PEGDE, 500 Da) and poly (ether imide) (PEI, 1800 Da) | Biocompatible, high throughput, easy to perform | Polydisperse, cannot achieve smaller size. (the diameter of their microrobot is 45-55 µm) | 23 |
| Conventional batch method | Magnetic nanoparticles, styrene | High throughput, easy to perform, low cost | Polydisperse, lack of magnetic anisotropy, non-biodegradable. | 24 |
| Droplet based microfluidic combine with controllable polymerization and magnetic programing | Magnetic nanoparticles, dextran/PEGDA | Biocompatible and biodegradable, high throughput, easy to perform, extensive choice of material, programming of overall structure and internal magnetic pattern, low cost | Cannot achieve arbitrary shape (limited to spheres, ellipsoids, and doublets) | This study |

**Table. S2.** Comparison of different fabrication techniques for magnetic microrobots.

**Supplementary References**

1. Soo Choi, H., Liu, W., Misra, P., Tanaka, E., Zimmer, J. P., Itty Ipe, B., Bawendi, M. G., Frangioni, J. V. Renal clearance of quantum dots. *Nat. Biotechnol.* **25**, 1165-1170 (2007).

2. Patra, J. K., Das, G., Fraceto, L. F., Campos, E. V. R., Rodriguez-Torres, M. d. P., Acosta-Torres, L. S., Diaz-Torres, L. A., Grillo, R., Swamy, M. K., Sharma, S., Habtemariam, S., Shin, H.-S. Nano based drug delivery systems: recent developments and future prospects. *J. Nanobiotechnology* **16**, 71 (2018).

3. Mirza, A. Z., Siddiqui, F. A. Nanomedicine and drug delivery: a mini review. *Int. Nano Lett.* **4**, 94 (2014).

4. Azandaryani, H. A., Kashanian, S., Jamshidnejad-Tosaramandani, T. Recent Insights into Effective Nanomaterials and Biomacromolecules Conjugation in Advanced Drug Targeting. *Curr. Pharm. Biotechnol.* **20**, 526-541 (2019).

5. Nazir, S., Hussain, T., Ayub, A., Rashid, U., MacRobert, A. J. Nanomaterials in combating cancer: Therapeutic applications and developments. *Nanomedicine: NBM* **10**, 19-34 (2014).

6. Gubernator, J. Active methods of drug loading into liposomes: recent strategies for stable drug entrapment and increased in vivo activity. *Expert Opin. Drug Deliv.* **8**, 565-580 (2011).

7. Hua, S., Wu, S. The use of lipid-based nanocarriers for targeted pain therapies. *Front. Pharmacol.* **4**, (2013).

8. Monteiro, N., Martins, A., Reis, R. L., Neves, N. M. Liposomes in tissue engineering and regenerative medicine. *J. R. Soc. Interface* **11**, 20140459 (2014).

9. Akbarzadeh, A., Rezaei-Sadabady, R., Davaran, S., Joo, S. W., Zarghami, N., Hanifehpour, Y., Samiei, M., Kouhi, M., Nejati-Koshki, K. Liposome: classification, preparation, and applications. *Nanoscale Res. Lett.* **8**, 102 (2013).

10. Heidarli, E., Dadashzadeh, S., Haeri, A. State of the Art of Stimuli-Responsive Liposomes for Cancer Therapy. *Iran J. Pharm. Res.* **16**, e127507 (2017).

11. Chargaff, E., West, R. The biological significance of the thromboplastic protein of blood. *J. Biol. Chem.* **166 1**, 189-197 (1946).

12. van Balkom, B. W. M., Gremmels, H., Giebel, B., Lim, S. K. Proteomic Signature of Mesenchymal Stromal Cell-Derived Small Extracellular Vesicles. *PROTEOMICS* **19**, 1800163 (2019).

13. Piffoux, M., Silva, A. K. A., Wilhelm, C., Gazeau, F., Tareste, D. Modification of Extracellular Vesicles by Fusion with Liposomes for the Design of Personalized Biogenic Drug Delivery Systems. *ACS Nano* **12**, 6830-6842 (2018).

14. Jabalee, J., Towle, R., Garnis, C. The Role of Extracellular Vesicles in Cancer: Cargo, Function, and Therapeutic Implications. *Cells* **7**, 93 (2018).

15. Teekamp, N., Van Dijk, F., Broesder, A., Evers, M., Zuidema, J., Steendam, R., Post, E., Hillebrands, J. L., Frijlink, H. W., Poelstra, K., Beljaars, L., Olinga, P., Hinrichs, W. L. J. Polymeric microspheres for the sustained release of a protein-based drug carrier targeting the PDGFβ-receptor in the fibrotic kidney. *Int. J. Pharm.* **534**, 229-236 (2017).

16. Costa, A. M. S., Alatorre-Meda, M., Oliveira, N. M., Mano, J. F. Biocompatible Polymeric Microparticles Produced by a Simple Biomimetic Approach. *Langmuir* **30**, 4535-4539 (2014).

17. Crucho, C. I. C., Barros, M. T. Formulation of functionalized PLGA polymeric nanoparticles for targeted drug delivery. *Polymer* **68**, 41-46 (2015).

18. Yang, Y., Bajaj, N., Xu, P., Ohn, K., Tsifansky, M. D., Yeo, Y. Development of highly porous large PLGA microparticles for pulmonary drug delivery. *Biomaterials* **30**, 1947-1953 (2009).

19. Wang, X., Qin, X.-H., Hu, C., Terzopoulou, A., Chen, X.-Z., Huang, T.-Y., Maniura-Weber, K., Pané, S., Nelson, B. J. 3D Printed Enzymatically Biodegradable Soft Helical Microswimmers. *Adv. Funct. Mater.* **28**, 1804107 (2018).

20. Tottori, S., Zhang, L., Qiu, F., Krawczyk, K. K., Franco-Obregón, A., Nelson, B. J. Magnetic Helical Micromachines: Fabrication, Controlled Swimming, and Cargo Transport. *Adv. Mater.* **24**, 811-816 (2012).

21. Safaee, S., Chen, R. Investigation of a Magnetic Field-Assisted Digital-Light-Processing Stereolithography for Functionally Graded Materials. *Procedia Manuf.* **34**, 731-737 (2019).

22. Alapan, Y., Bozuyuk, U., Erkoc, P., Karacakol, A. C., Sitti, M. Multifunctional surface microrollers for targeted cargo delivery in physiological blood flow. *Sci. Robot.* **5**, eaba5726 (2020).

23. Xie, M., Zhang, W., Fan, C., Wu, C., Feng, Q., Wu, J., Li, Y., Gao, R., Li, Z., Wang, Q., Cheng, Y., He, B. Bioinspired Soft Microrobots with Precise Magneto-Collective Control for Microvascular Thrombolysis. *Adv. Mater.* **32**, 2000366 (2020).

24. Taddei, C., Sansone, L., Ausanio, G., Iannotti, V., Pepe, G. P., Giordano, M., Serra, C. A. Fabrication of polystyrene-encapsulated magnetic iron oxide nanoparticles via batch and microfluidic-assisted production. *Colloid Polym. Sci.* **297**, 861-870 (2019).
